# A flexible liquid metal magnetohydrodynamic pump for soft robotic systems

**DOI:** 10.1038/s41467-026-72798-7

**Published:** 2026-05-12

**Authors:** Saba Firouznia, Christian Romero, Ciqun Xu, Lihaoya Tan, Andrew Stinchcombe, Martin Garrad, Andrew Conn, Hemma Philamore, Jonathan Rossiter

**Affiliations:** School of Engineering Mathematics and Technology, and Bristol Robotics Laboratory, University of Bristol, Bristol, UK

**Keywords:** Mechanical engineering, Engineering

## Abstract

Fluidic soft actuators enable safe, adaptable human-machine applications, but suffer from shortcomings of portability and autonomy. Herein, we introduce a liquid metal droplet based fluidic soft pump, which is lightweight (0.2 g) and compact (0.086 cm³), delivering specific pressure (18.6 − 34.88 GPa m⁻³) and specific flow rate (38.4 − 49.29 kL min⁻¹ m⁻³), surpassing existing soft and conventional pumps, and operating at low voltage ( < 1 V). Electrical energy is converted into fluid motion through the principle of magnetohydrodynamics, which harnesses the Lorentz electro-magnetic force through the liquid metal. We present the optimization of the pump design, evaluate its fundamental characteristics, and demonstrate its integration with a range of soft robotic systems. The versatility of the pump, including powering soft actuators, transferring chemical energy, and generating diverse code patterns, illustrates the potential for the development of future advanced robotic systems.

## Abstract (web summary)

A liquid metal magnetohydrodynamic pump, LIMA, demonstrates enhanced portability and efficiency for soft robotics. Utilizing low voltage and optimized design, it achieves high flow rates and pressures, enabling versatile applications in fluid propulsion and information transfer.

## Introduction

Researchers and engineers are increasingly interested in developing soft robots that can emulate biological functions, such as skin-like perception and muscle-like actuation^1–4^. Soft robots offer more adaptable and safer ways for humans and machines to interact compared to traditional rigid robots, and show great potential to meet global challenges across environmental, healthcare, and industrial applications. Various soft actuation techniques have been explored to achieve movement in soft robots^5–7^, including pressure^8–10^, thermal^11,12^, magnetic fields^13,14^, electric fields^15^, light^16^, combustion^17,18^, and phase transition^19^.

Fluidic elastomer actuators (FEAs) are attractive for use in soft robotics due to their inherent compliance, low cost, and ability to articulate with multiple degrees of freedom into complex forms^2,20^. These actuators consist of expandable fluidic chambers embedded within a highly deformable elastomer, allowing for bending, twisting, tensile, compressive, and contractile modalities. They have been successfully utilized in applications ranging from manipulation^21,22^ and locomotion^23^ to wearables^24^ and biomedical applications^25,26^.

However, the portability and application of traditional fluidic soft actuators, especially in untethered applications, are hindered by the requirement for external bulky compressors or pumps^27,28^. The conventional setup of a single centralized fluidic compressor with multiple distributed fluidic actuators is limited due to the scaling of pressure losses as the fluid supply channels become thinner and longer. To address these limitations, miniature piezoelectric, electromagnetic^29^, and chemical combustion pumps have been developed^18^, which have the potential to decentralize the pressure supply and compensate for pressure losses. However, their rigid nature compromises compliance and advanced behaviors such as computational morphology, ultimately leading to overcomplicated system designs^30^.

To enhance the performance of soft robots, it would be advantageous to employ small pumps, which possess mechanical properties akin to those of FEAs, and which could be distributed throughout the robot’s volume. This would yield benefits, including situating the pumps closer to actuators for better efficiency and coupling multiple small pumps to achieve faster and stronger actuation where needed, while retaining the intrinsic compliance of the soft robot. Several pumps constructed entirely of compliant materials have been developed, including those developed by Cacciucolo et al.^31^ and Diteesawat et al.^32^, as well as others based on soft displacement^33–37^ or rotary action^38–40^. However, all employ a specific operational fluid or require high voltages ( > 1 kV), which limits their practical application, underscoring the need for a small embeddable compliant pump that can provide high flow rate and pressure with any fluid (i.e., air and water) and without the need for high voltage electronics and insulation.

Here we present a liquid metal magnetohydrodynamic pump (LIMA), which is driven by the electric-current-induced Lorentz force within a small droplet ( < 0.1 mL) of liquid metal. The liquid metal provides multiple key features that enable high performance of the LIMA pump: 1. The high surface tension of the liquid metal creates a good seal against the walls of the pumping chamber; 2. The liquid metal acts as a mobile charge carrier with extremely low resistance to motion; 3. The extremely high conductivity of liquid metal (3.4 × 10^6^ S m^−1^)^41,42^ enables the passing of high currents (8 A) through a 100 µm thickness droplet at very low driving voltages ( ~ 0.1 V), thereby generating high Lorentz forces which are readily transferred to the pumped liquid; 4. The liquid metal is deformable under external load (e.g., if the pump is flexed), with its high surface tension (624 × 10^−3^ N m^−1^) resisting breakup and resulting in a robust, soft pump; 5. The LIMA pump is very lightweight (0.2 g) and compact (0.086 cm^3^), silent, bidirectional and controlled by simply changing the magnitude and polarity of applied current; 6. The fluidic nature of the liquid metal, which internally reconfigures at each pump cycle through internal flows, mitigates the local stress concentrations that cause breakdowns in traditional rigid pumps (e.g., impeller or piston)^43^.

In recent years, liquid metals, including alloys such as eutectic GaIn (EGaIn, 75% gallium and 25% indium)^44,45^ and Galinstan (68.5% gallium, 21.5% indium, and 10% tin)^46^, have received increasing research attention. These liquid metals exhibit desirable properties such as high electrical conductivity, high density, high surface tension, low vapor pressure, and low toxicity compared to traditional counterparts such as mercury^46^. As a result, they are ideal for use in a wide range of applications, including soft electronics^44^, stretchable components^47^, MEMS devices^48^, and nanotechnology-enabled applications^49^. This study explores the use of liquid metal as the core of a pump that converts electrical energy into mechanical energy utilizing magnetohydrodynamics.

Previous research has explored liquid-based microscale soft pumps, but achieving sufficient pressure and flow rate to power macroscale actuators and soft robots is still a challenge. Our study demonstrates the soft microscale LIMA pump as a power source for a soft macroscale arthropod-inspired leg^50^, generating reciprocal motion through the inflation of its soft joint. We also highlight the LIMA pump’s unique ability to manage dual fluids with its differential outlet system, as seen through the conveyance of red and blue-dyed fluids in a soft 3D-printed display bracelet. This feature not only allows for traditional fluid conveyance and color presentation but also serves as an innovative means of transferring chemical energy to actuate a soft haptic actuator.

**Fig. 1:**
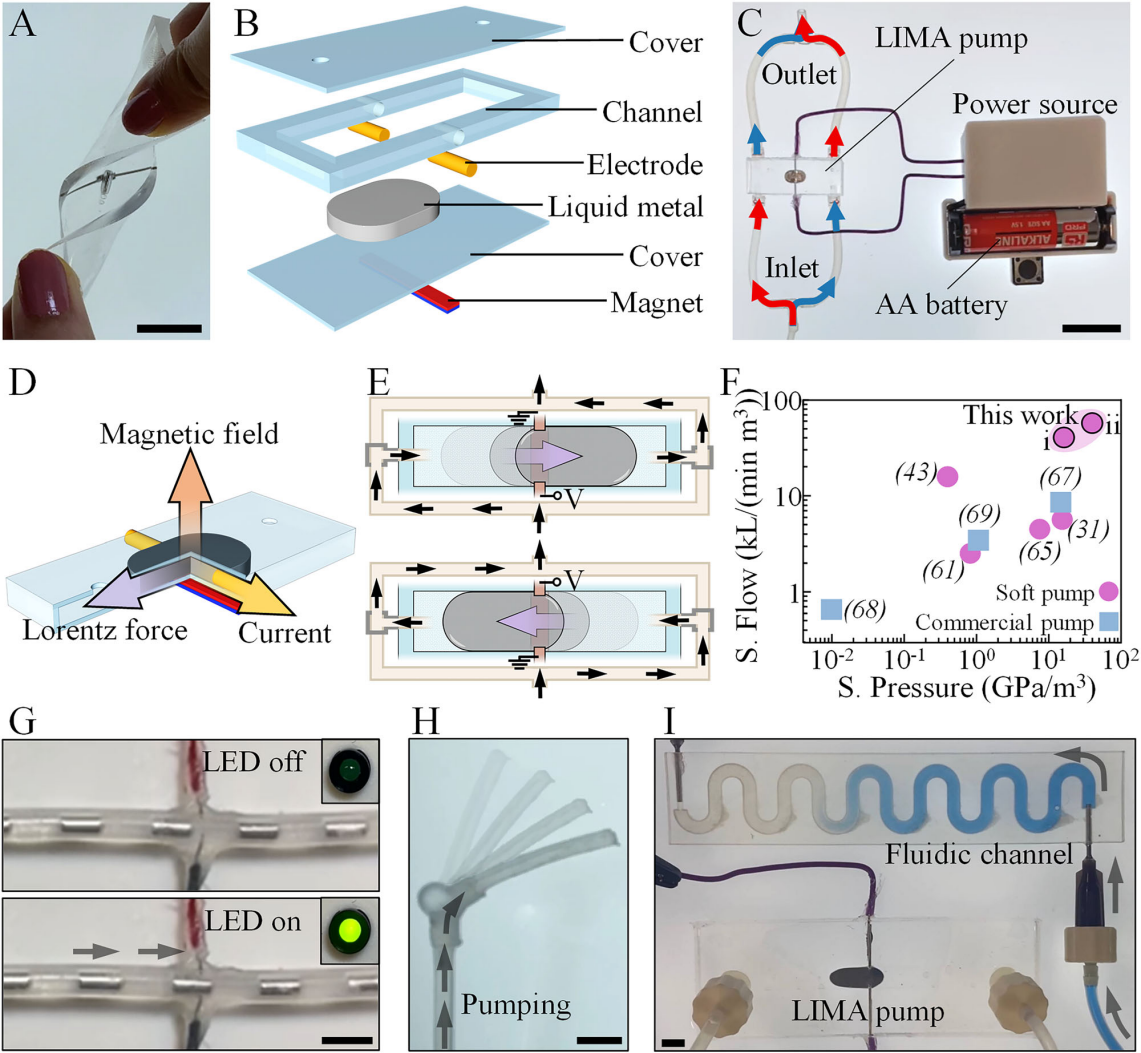
Principal concepts of the LIMA pump. **A** Flexibility of the LIMA pump. (see Supplementary Movie 1 for full demonstration). **B** Exploded diagram of the deformable pump showing its components. **C** Demonstrating the portability and compactness of the LIMA pump. **D** The right-hand rule linking magnetic field, current density and Lorentz force acting on the liquid metal. **E** Operating principle of the pump. **F** Comparison of specific flow rate versus specific pressure of LIMA pump (solenoid (ii) and passive check valves (i)) and other soft and commercial pumps. The detailed data are presented in Table S1. **G** Control and information coding capabilities of the LIMA pump. **H** Soft actuator driven by the LIMA pump pressure. **I** Deformable LIMA pump-driven fluid flow and color display. Scale bars are 5 mm.

The growing demand and enthusiasm for soft robotics systems have motivated the development of soft computational systems to support advances in soft bodies. These systems require mechanisms for generating and transmitting control signals. Rothemund et al.^51^ and Drotman et al.^52^ demonstrated that periodic motion can be achieved by using a constant source of compressed fluid and the logic output from a soft bistable valve system. Garrad et al.^53^ demonstrated soft matter computers to encode information and integrate computation directly into soft materials. Within this system, our LIMA pump demonstrated an elementary signal encoding via controlled fluid distribution, while simultaneously transferring simple information and energy around a soft body. We generate varied digital code patterns by controlling the pump, with analog quantities optionally encoded in the duty cycle. Here, simple information is carried by the spatial and temporal structure of fluid pulses as they propagate through the body, analogous to biological signaling processes in which transported chemical messengers elicit a response only when detected by an appropriate receptor. This information is transmitted within the soft robot’s body at variable speeds to receptors, which then activate the distal actuators. In the present work, these transmitted patterns are converted to electrical signals to drive secondary elements (e.g., LEDs or shape-memory alloys), serving as a proof of concept of readable outputs of the underlying fluidic signal without requiring direct fluidic actuation at the output site. In future implementations, similar principles could also be realized using distinguishable immiscible fluids (for example, color-coded phases) to carry information through the body. While these examples show the system’s versatility and suggest directions for further study, the primary role of the LIMA pump is fluid propulsion, with these signal-based behaviors representing secondary capabilities enabled by its controllable flow output. The LIMA pump is expected to enable a wide range of softer, simpler, and more intelligent soft-robotic systems (Fig. 1).

## Results

### Concept of LIMA pump

The LIMA pump exploits the principle of magnetohydrodynamics. It consists of a liquid metal droplet surrounded by sodium hydroxide (1 M) suspended in a soft microchannel between a pair of electrodes (Fig. 1B–D). An alternating voltage on the order of millivolts is applied to the electrodes (Fig. S1), inducing a current in the liquid metal. A small permanent magnet (dimensions 5 mm × 1.5 mm × 1 mm as shown in Figs. S2, S3) is placed underneath the channel to create a local magnetic field perpendicular to the current. The moving charges within the liquid metal pass through the magnetic field and generate a Lorentz force (Fig. 1D). This force, the direction of which is governed by the direction of the current, acts upon the liquid metal and causes it to move back and forth, generating a fluid flow and a pumping action. Due to the placement and size of the small magnet in the LIMA, it has a minimal effect on the pump’s deformability (as shown in Fig. 1A).

The LIMA pump is fabricated by plasma-bonding two polydimethylsiloxane (PDMS) layers with a microchannel (Fig. S4) molded into the interface between them (Fig. 1B). Two identical pin electrodes are inserted into the microchannel orthogonally from both sides and sealed (Fig. 1B). The microchannel is first filled with NaOH and subsequently, a small droplet of liquid metal is injected into the channel to connect the two electrodes. The NaOH dissolves oxidation species from the surface of the liquid metal^54,55^, allowing the metal to function as a fluid. Due to chemical wetting, the droplets anchor at the electrode after contact, maintaining electrical contact, and generating a small restoring force to the equilibrium position where the droplet is symmetrical about the electrodes.

Two three-way valves are connected to the ends of the channel, rectifying pressure and flow cycle, and thereby generating a continuous one-way pumping action (Figs. 1E, S5 and S6). To optimize pumping flow rate and pressure, we consider the related fields of electromagnetism and hydrodynamics. According to Faraday’s law, the total Lorentz force applied to the liquid metal is given by Eq. (1). Assuming a uniform distribution of current between the electrodes, the expression for static pressure head can be simplified as Eq. (2)^56^,

1 $$F={\int }_{0}^{W}{BIdy}$$

2 $$\triangle P=\frac{{BI}}{h}$$

where *B* represents the magnetic field, *I* is the electric current through the liquid metal, Δ*P* is the average static pressure head, *h* is the height of the channel, and *W* is the width of the channel (Fig. S7). Equation (2) implies that a reduction in channel height yields an elevation in the pressure generated by the system. However, reducing channel height also leads to an increase in flow resistance, reducing the flow rate. Prior work investigating the MHD principle has focused on DC and channel heights of 1 mm and above^57^. Advancing the state-of-the-art, in this research, we focus on sub-millimeter channel heights and exploit AC drive, aiming to attain the optimal performance of our soft deformable pump.

### Performance characterization

To investigate the influence of different channel heights on the performance characteristics of the LIMA pump, a series of experiments were conducted using four distinct channel heights. The pressure and flow rate produced by the LIMA pump were measured as we varied the current and length of the liquid metal droplet. During the characterization of the LIMA pump, two infrared sensors were employed to track the endpoints of the liquid metal during each stroke and to precisely control the current in the pump. The stroke amplitude is dependent on the length of the droplet and the position of the sensors.

**Fig. 2:**
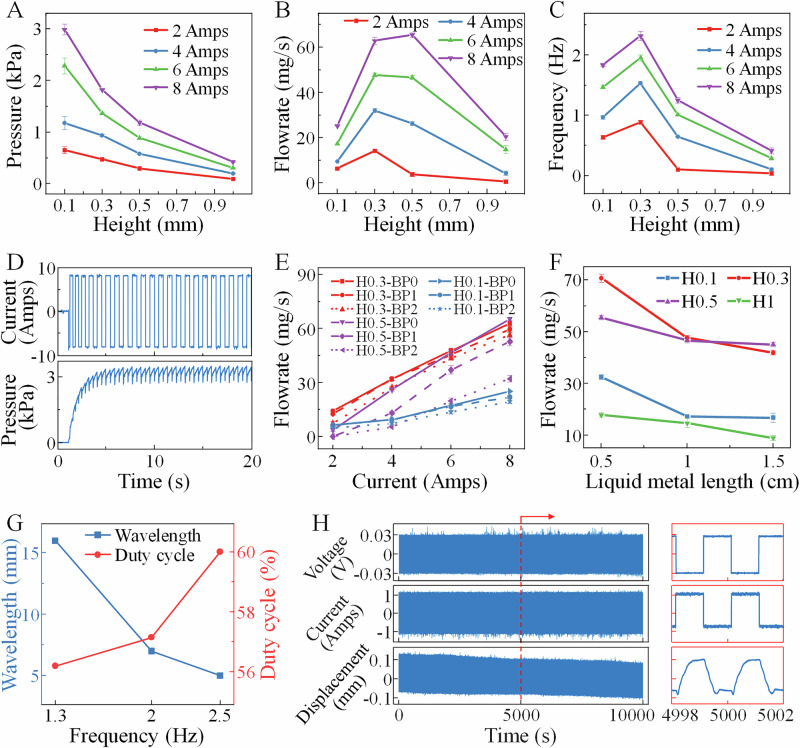
Performance characterization of the LIMA pump. **A** Pressure versus channel height for different currents. Error bars represent the standard deviation of three measurements. **B** Flow rate versus channel height for different currents. **C** Frequency versus channel height for different currents. **D** Ramp-up response of the pump. **E** Flowrate versus current for different back pressures for different channel heights (mm) **F** Flowrate versus liquid metal length for different channel heights (mm) for a current of 6 Amps. **G** Wavelength and duty cycle of the code generated by different frequencies of the pump for channel height 0.3 mm. **H** Cyclic test on the LIMA pump, i. voltage applied, ii. current applied and iii. The displacement of NaOH in the reservoir.

Figure 2A plots the pressure generated by the LIMA pump for a range of currents and channel heights against closed output. The data shows that higher applied currents led to higher pumping pressures. The detailed explanation of the characterization apparatus is included in the materials and methods. The maximum pressure levels were achieved when the channel height was at its lowest (0.1 mm), a behavior consistent with the governing Eq. (2). The highest flowrate (Fig. 2B) values were induced using the sample with a channel height of 0.3 mm (for all currents up to 8 Amps), which coincides with the highest emergent frequency achieved from the infrared sensor feedback (Fig. 2C). Note that increasing channel height results in reduced hydrodynamic resistance; however, it also entails a decrease in the magnetic field strength at the center of the liquid metal (due to field divergence), coupled with an increase in the mass of the liquid metal (given a constant liquid metal droplet length) and reduced sealing pressure on the walls of the microchannel from reduction in Laplace pressure. These combined factors contribute to the observed optimal performance at a channel height of 0.3 mm. Figure 2D illustrates a rapid rise in pressure from the point of pump activation. This upward trajectory persists until reaching the peak blocking pressure at approximately 5 s.

A standard way of evaluating the performance of the LIMA pump is by characterizing the flow rate achieved under different back pressures at various electric currents. Figure 2E shows that without back pressure (BP0), the flow rate increases as the channel height increases from 0.1 mm to 0.3 mm across all current values. By increasing the channel height from 0.3 mm to 0.5 mm, it is observed that the flow rates decrease for most of the currents applied, and at currents higher than 6 Amps, the flow rates become similar to those recorded at 0.3 mm. This result closely resembles the measured flow rates with back pressure of 0.25 kPa and 0.5 kPa referred to as BP1 and BP2, respectively. The findings align with the results shown in Fig. 2B, C, demonstrating that the LIMA pump with a channel height of 0.3 mm generates the highest flow rate in both cases, with back pressure and without back pressure. A separate plot showing the flow rate against different back pressures for each channel height is also presented in Fig. S8 for further clarification. Another design aspect of the LIMA pump is its adjustable droplet size. By simply increasing the length of the liquid metal droplet from 0.5 cm to 1.5 cm, we observed a consistent decrease in flow rate across all channel heights (Fig. 2F). This result can be due to the interplay between the droplet size and the Lorentz force driving it. As we increase the size of the droplet, the mass increases; however, the force responsible remains constant. This imbalance decreases droplet velocity and the pump’s flow rate.

Unlike many previous soft pumps, the LIMA pump operates at an extremely low voltage ( < 1 V) (Fig. S9) and demonstrates low power consumption, with a maximum of 0.8 W at 0.1 V and 8 Amps. According to the Lorentz force Eq. (1), the induced force can be enhanced by either increasing the current *I* or strengthening the magnetic field *B*. To explore this, we simulated stacked LIMA configurations with and without a closed magnetic circuit (illustrated in Fig. S10). COMSOL simulation results reveal that using a closed magnetic circuit increases the magnetic field strength by more than 300% while reducing power consumption by a factor of nine to just 0.07 W. Additionally, we experimentally validated the performance enhancement using two magnets, demonstrating an increase in fluidic output and achieving an approximately 400% improvement in efficiency (Figs. S10–S13).

Furthermore, cyclic testing was conducted over an experimental time of 10,000 s at a frequency of 0.5 Hz (Fig. 2H). During this test, a square wave current of ±1 Amp was applied to the LIMA pump with a channel height of 0.3 mm. The height of sodium hydroxide (NaOH) in a reservoir was monitored using a laser displacement meter. In addition, the LIMA pump operated continuously for 48,600 s (13.5 h) at 1 Hz, accumulating 48,600 cycles. Over this extended duration, a minor change in actuation amplitude was observed, attributed to minimal oxidation of the liquid metal droplet, as shown in Fig. S14.

**Fig. 3:**
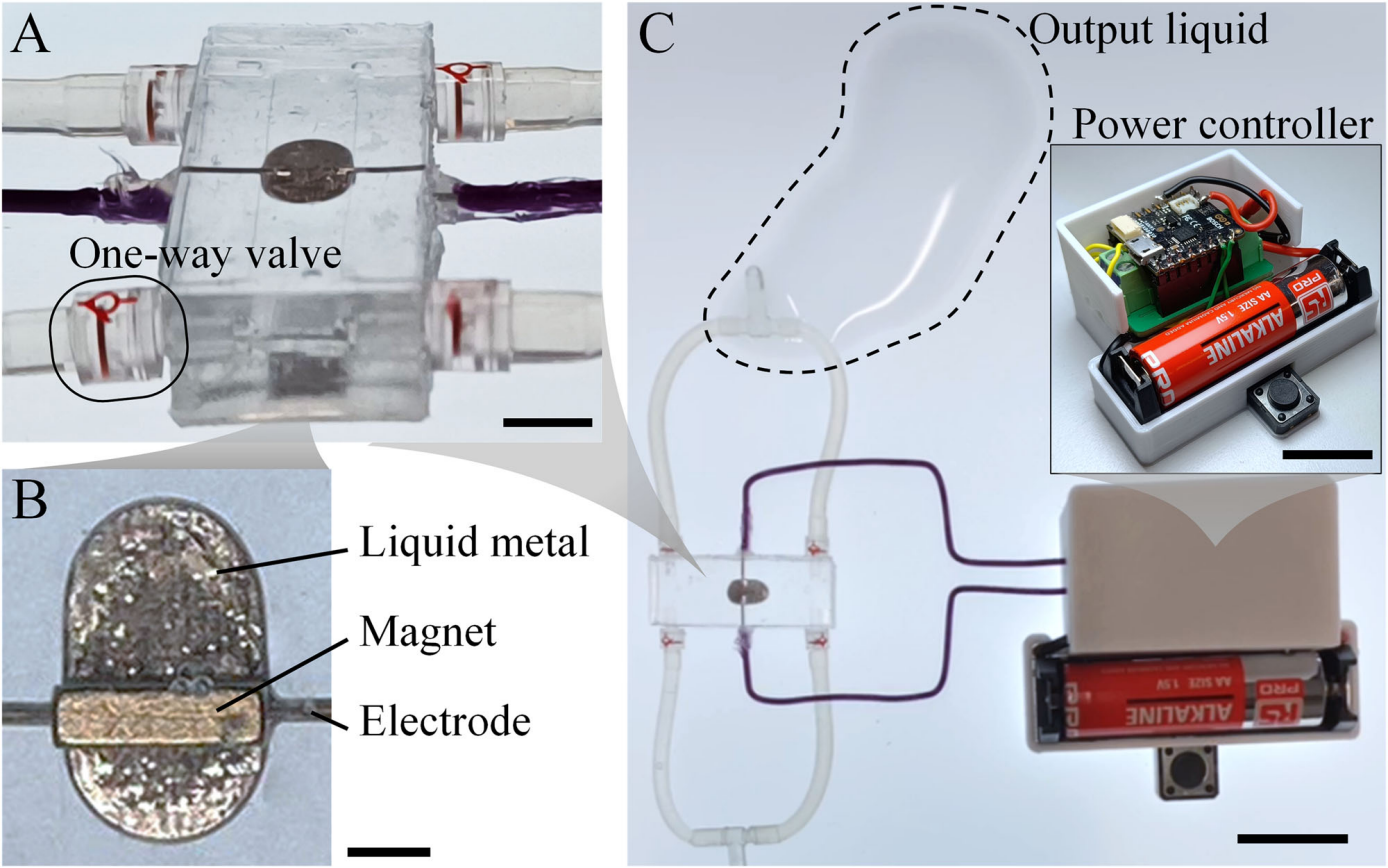
A portable LIMA pump system. **A** Picture of the LIMA pump integrated with four one-way valves. Scale bar is 5 mm. **B** Enlarged view of the active section of the pump. Scale bar is 2 mm. **C** The final frame of the Supplementary Movie 9, which shows the overview of the LIMA pump system and the displaced fluid from the output. The inserted picture shows the power controller of the pump, composed of an electronic controller, an AA battery, and a switch. Scale bar is 2 cm.

Moreover, we evaluated the system’s performance by replacing the two active solenoid valves with four (non-optimal) passive check valves. Compared to the original setup, using check valves caused a small performance reduction: the maximum flow rate dropped to 3.3 mL/min and the maximum pressure to 1.6 kPa. This decrease occurred because check valves require pressure to open, as detailed in Figs. S15, S16. As shown in Fig. 3, the LIMA pump is implemented as a compact, self-contained system powered by AA batteries with integrated control electronics, enabling stand-alone and portable operation. The fabrication material cost of the actuation unit of the LIMA pump prototype is less than 50 UK pence (63 US cents), and this cost can be significantly reduced through mass production.

### LIMA capabilities demonstration

**Fig. 4:**
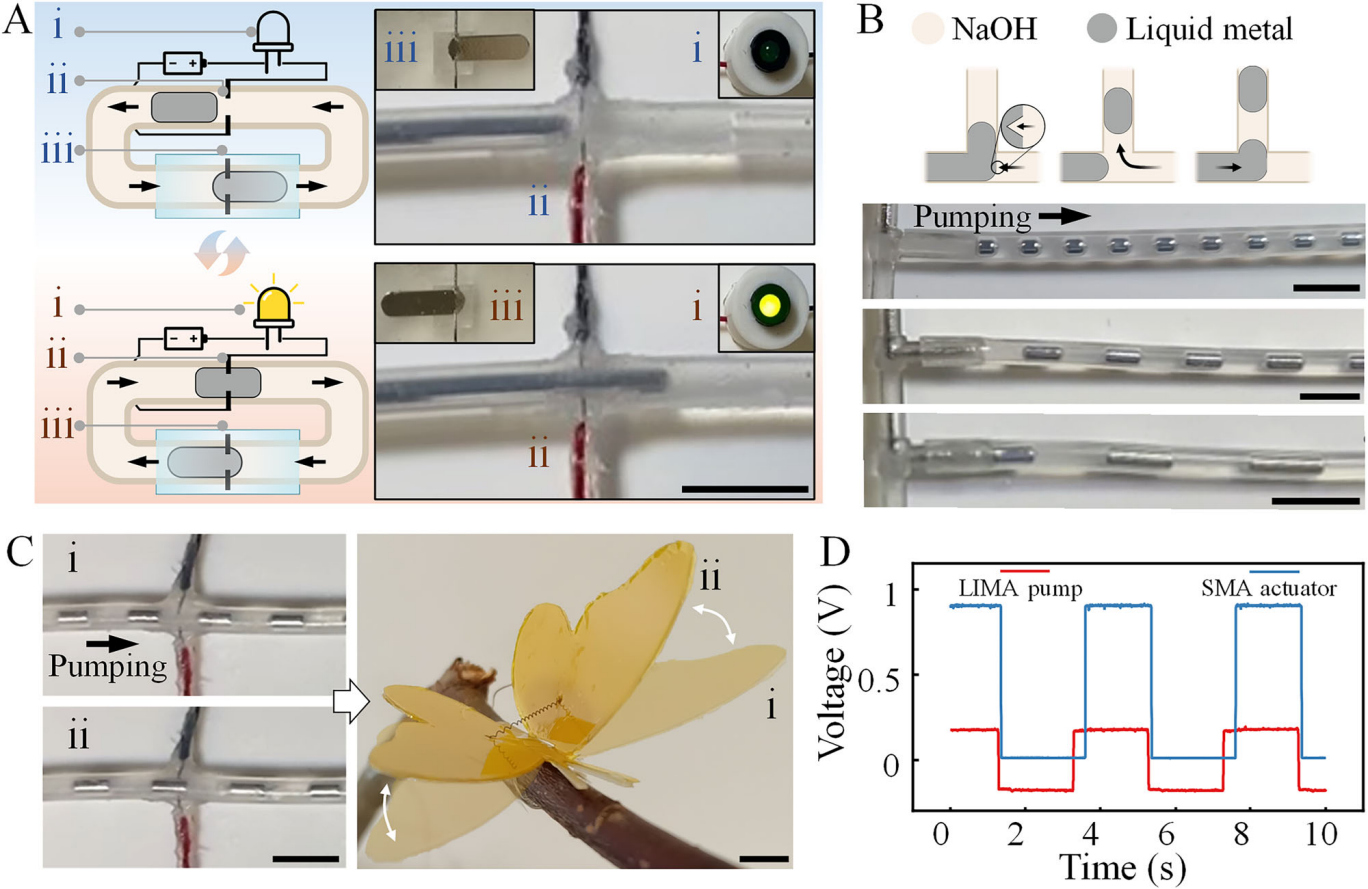
Control capabilities of the LIMA pump. **A** diagram and pictures of using the liquid metal in the micro channel as a switch (soft matter receptor) (ii) to control a remote LED (i) by valveless LIMA pump (iii). **B** diagram of code pattern creation by intermixing liquid metal and NaOH (top) and three patterns of different wavelength and duty cycles created by the LIMA pump (bottom). (see Supplementary Movie 2 for full demonstration). **C** the code generated by the LIMA pump flapping remote butterfly wings via a soft matter receptor. **D** Voltage of the LIMA pump and SMA actuators driving the butterfly wings. Scale bars are 5 mm.

A series of experiments were conducted to evaluate the practical applicability of the LIMA pump in real-world scenarios. First, we investigated the capability of the LIMA pump to facilitate piston-like motions without the need for valves. These motions were employed to control remote switches in an electronic circuit, wherein the frequency of the liquid metal’s movement within the LIMA pump served as a determinant for the ON-OFF frequency of an LED (Fig. 4A). Here, the liquid metal droplet in the LIMA pump moves an aqueous solution in a tube, which in turn moves a remote droplet of liquid metal. This remote droplet bridges two electrode contacts in the tube, alternately connecting and disconnecting the LED from its power source (Fig. 4A), implementing a soft matter receptor. This demonstrates the simplest transmission of information within the body of a soft robot and can be utilized in a variety of soft matter computer applications^53^. Next, we employed the differential outlet system to generate a coding stream of liquid metal/NaOH droplets. The differential outlet system was implemented where one outlet (one side of the LIMA pump) pumped liquid metal, creating a conductive fluid pathway, while the second outlet (connected to the other side of the LIMA pump) pumped sodium hydroxide, establishing a non-conducting fluid pathway (Fig. 4B). The two outlets were connected at a T junction to permit the two immiscible fluids to mix (Fig. 3C, bottom). By modulating the frequency of the pump, it could generate a stream of intermixed regions of liquid metal and sodium hydroxide. The wavelength and duty cycle of the liquid metal/NaOH stream varied with frequency, as shown in Figs. 4B, 2G. The duty cycle (defined as 100% for pure NaOH and 0% for pure liquid metal) exceeded 50% due to the time and pressure required for the NaOH to separate a droplet of liquid metal from the liquid metal supply (Fig. 4B.top). This mechanism can be used to tune the frequency that the switching system will use to alternate between the ON and OFF states, with a neglectable change in duty cycle (Fig. 2G) (see Supplementary Movie 2 for full demonstration). Encoded streams of liquid metal/NaOH were generated and delivered by the LIMA pump through a tube to a remote electrical switch receptor, which was connected to a shape memory alloy-actuated butterfly robot (Fig. 4C). When a liquid metal droplet bridged the receptor, the butterfly’s wings elevated; when a NaOH droplet passed through the receptor, the butterfly’s wings relaxed. Modulating the rate and duty cycle of the generated vascular code (liquid metal/NaOH) enabled control over the wing flapping pattern (Fig. 4D, Supplementary Movie 3). This demonstration provides a visual readout of both the fluidic frequency and the conductive pattern produced by the pump.

**Fig. 5:**
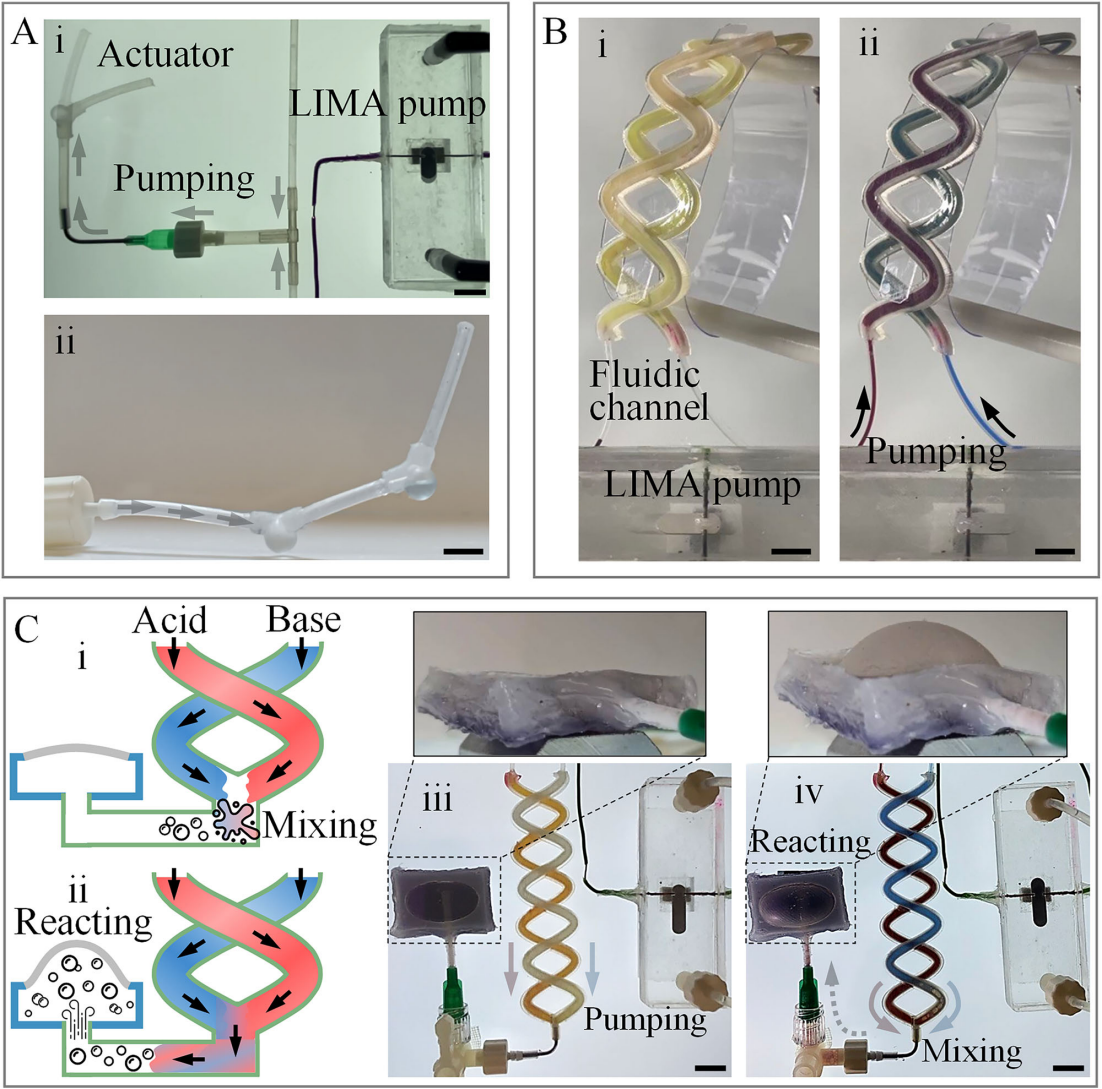
LIMA pump for hydraulic actuation and dual liquid pumping. **A** Arthropod-inspired actuators with inflatable joints. i: single joint; ii: double joint. **B** A color-changing bracelet powered by LIMA pump. i: before color change, ii: color changed in 28 s. **C** Transfer of chemical reactions via dual liquid pumping. i: pumping citric acid (acid, red) and sodium bicarbonate (base, blue) into the fluidic channels; ii: the reaction begins when the acid and base are mixed, resulting in gas generation; iii: experimental setup before liquid pumping; iv: experimental setup during the chemical reaction, in which the pneumatic haptic actuator is stimulated. Scale bars are 5 mm.

To demonstrate the application of the LIMA pump as a fluidic power source for macroscale soft robots, we used the LIMA pump to drive various compliant fluidic actuators, a practice commonly integrated with traditional rigid pumps or compressors. Figure 5A depicts one-joint (i) and two-joint (i) pneumatic legs inspired by the morphology of arthropod joints^50^ powered by the bidirectional LIMA pump, which generates reciprocal motion through the inflation/deflation of its soft joint(s) (see Supplementary Movies 4, 5). The one-joint actuator exhibits a deflection exceeding 45° relative to its initial position. These actuators form the basis for developing subsequent generations of soft robots, combining the durability, substantial deformation capacity, and adaptability of fluidic actuators with the portability inherent in an integrated system that eliminates the need for external compressors.

Next, we demonstrated the unique capability of the LIMA pump to simultaneously pump different fluids, enabled by its differential outlet system and integrated air gap (Fig. S17). We fabricated a flat-helix-structured soft bracelet with two fluidic channels, each connected to an outlet of the LIMA pump via tubing. (Fig. 5B). By pumping two differently colored liquids into the bracelet, the bracelet’s color can be dynamically changed in 28 s (Supporting Information Fig. S18, Video S6), revealing the potential for the development of customizable fashion wearables. Additionally, the dual liquid pumping system can be utilized to transfer chemical reactions. As shown in Fig. 5C, a red-dyed acid (citric acid) and a blue-dyed alkali (sodium bicarbonate solution) are simultaneously injected into the helical-structured fluidic channels using a LIMA pump, and they mix at the channel outlet. The outlet is connected to a pneumatic haptic actuator. A neutralization reaction is initiated when the two liquids are mixed, producing CO_2_ gas that subsequently inflates the pneumatic actuator. This demonstration illustrates that the LIMA pump can serve not only as a fluid transport and color display system, but also as an approach for transferring chemical energy to energize remote soft actuators.

**Fig. 6:**
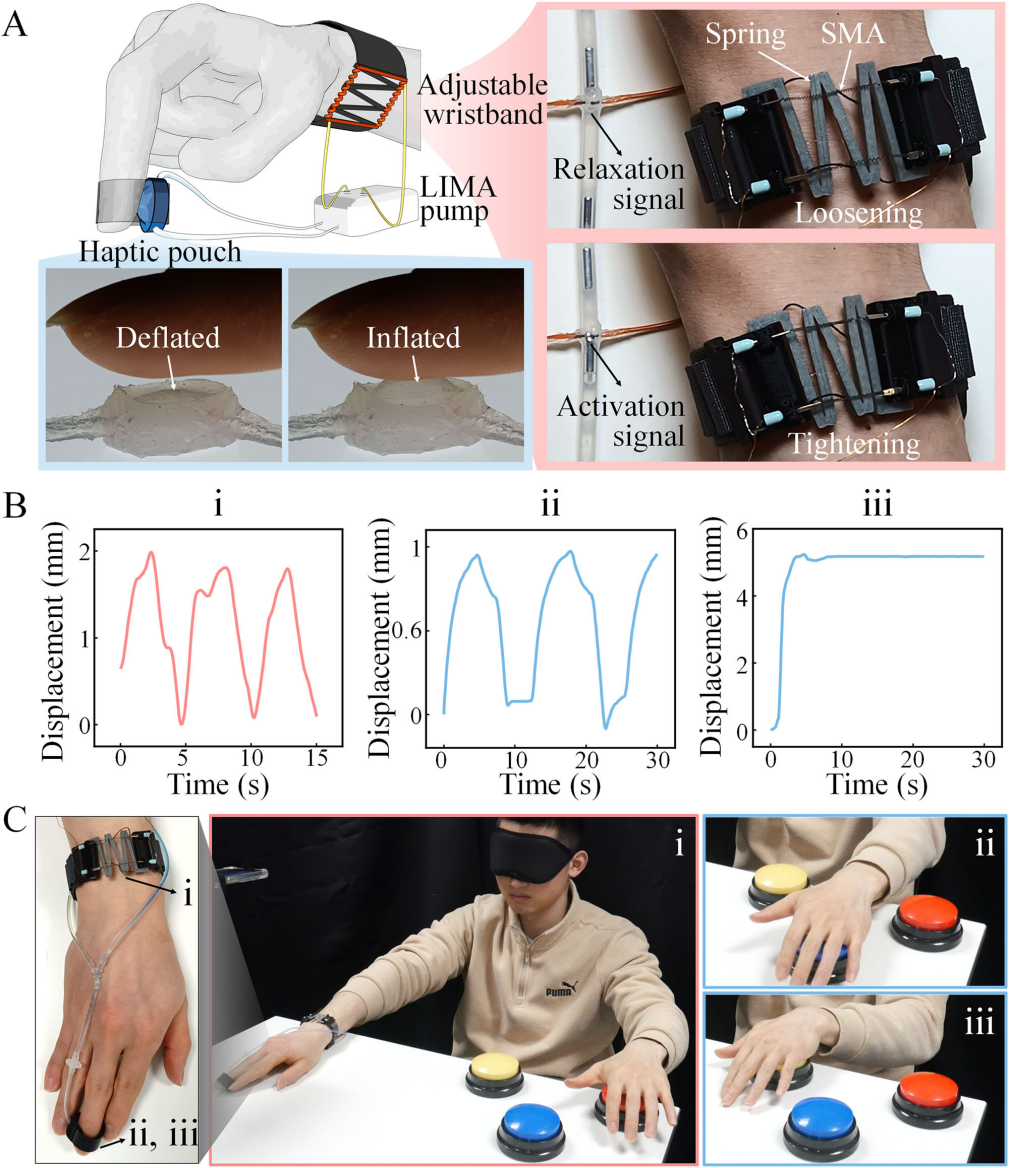
Demonstration of a single unit of LIMA pump enabling hydraulic pumping, information encoding, and chemical energy delivery modes. **A** Diagram of the wearable device: (Left) a haptic actuator worn on the finger delivering a gentle pressure cue via hydraulic actuation; and the same actuator delivering a stronger pressure cue due to gas generation from the chemical reaction. (Right) a wristband actuated by shape memory alloys applying circumferential squeezing force to the user’s wrist via coded signals generated by the LIMA pump. **B** Displacement of the tactile actuators corresponding to each mode: (i) wristband’s displacement in the information encoding mode, (ii) haptic actuator’s displacement in hydraulic mode, and (iii) haptic actuator’s displacement in chemical energy delivery mode. **C** Top view of the wearable device worn by a participant. (i) Participant sensing the first mode and pressing the red button; accordingly, (ii) participant sensing the second mode and pressing the blue button; (iii) participant sensing the third mode and pressing the yellow button.

Furthermore, we created a tactilewearable device as a proof-of-concept that uses a single LIMA pump unit to deliver various tactile sensations (Figs. 6, S19). This was achieved by information transfer, hydraulic actuation, and chemical energy delivery. Specifically, the prototype includes a wristband that applies circumferential force to the user’s wrist using integrated shape memory alloy (SMA) actuators. The code generated by the LIMA pump controls SMA actuation, demonstrating the simple programmable electrical switching capability of our system, as shown in Fig. 6A. The preliminary wearable device also includes a haptic actuator that, when worn on the finger, can deliver a gentle pressure sensation when driven by the hydraulic mode of the LIMA pump and a stronger sensation when driven by the system’s chemical energy delivery mode, as shown in Fig. 6A. The chemical energy delivery mode in its current implementation is single-use, which could be seen as an alert sensation. The SMA and haptic displacements regarding the three sensations are shown in Fig. 6B. To demonstrate the effectiveness of this system, we fitted the wearable prototype to the right hand of a blindfolded participant. The user was asked to press the button corresponding to each sensation they felt, based on the button’s position relative to their left hand, as shown in Fig. 6C. The user successfully identified the three tactile modes, as shown in Supplementary Movie 8. In this preliminary demonstration, to avoid sensory overlap, the timing of each mode was controlled manually using three-way valves (Fig. S19). We emphasize that this work does not constitute a validated human–robot interaction study, but rather presents an initial prototype demonstration. This kind of soft wearable haptic system can potentially provide more effective, localized, and expressive touch interactions than conventional vibrating actuators, enabling richer forms of communication, interaction, and immersion in compact wearable formats^58–60^. Future work will focus on system optimization and evaluation with a larger cohort of participants.

## Discussion

In this work, we demonstrate the LIMA pump, a magnetohydrodynamics-based, micro-scale, soft, deformable pump for generating and controlling fluid flow. We investigated the influence of channel height, liquid metal length, and current on output flow rate and pressure. We demonstrated the performance of the LIMA pump in generating bi-directional flow up to 70 mg s^−1^ and a maximum blocked pressure of 3 kPa. The LIMA pump is extremely light (0.2 g) and small in size (0.086 cm^3^). It exhibits a specific pressure of 34.88 GPa m^−3^ and a specific flow rate of 49.29 kl min^−1^ m^−3^, which is higher than previous soft pumps and commercial pumps as presented in Fig. 1F and Table S1. Moreover, in contrast to many previous soft pumps, the LIMA pump is driven by extremely low voltage ( < 1 V) and exhibits a low power consumption (maximum 0.8 W at 0.1 V and 8 Amps).

In addition to its primary function as a fluid displacement pump, our study showcases the remarkable capacity of this device for information transfer and control, effectively serving as a core component of an embodied soft computing system. Furthermore, the pump exhibits a unique dual-fluid handling capability, enabling it to pump two distinct fluids and thereby generate coded information for transport through a vascular system, while simultaneously delivering hydraulic and (optionally) chemical energy. This distinctive combination of functionalities sets the LIMA soft pump apart from prior works in the field, effectively integrating bio-inspired elements of both information processing (brain and central nervous system) and pumping (heart) within a single compact design, thereby positioning it as a potential core element for future soft robotic systems.

Building upon the excellent performance demonstrated by the LIMA pump, there exist several avenues for optimizing its design and functionality. Further exploration of different electrolyte solutions, such as increasing the molarity of sodium hydroxide (NaOH), may further reduce friction between the liquid metal and the channel and consequently minimize system losses. Scaling through series and parallel configurations allows fluidic systems to balance pressure and flow rate, enabling fluidic power scaling with system size.

In addition, evaluating different channel designs and electrode placements may enable self-sensing capabilities for the pump, automatically switching the current polarity as the liquid metal droplet reaches its stroke limits (Fig. S20). Furthermore, advances in high-energy-density low-voltage batteries are well-suited to advancing the capabilities of the LIMA pump and further widening its considerable promise in applications in bio-inspired robotics and smart microfluidics.

## Methods

### Manufacture of the LIMA pump

The deformable LIMA pump consists of a microfluidic channel filled with sodium hydroxide and a droplet of liquid metal embedded inside it, two electrodes and a small magnet underneath the droplet. The microfluidic channel includes a top and bottom layer fabricated from polydimethylsiloxane (PDMS) and bonded together using a Zepto plasma surface treatment machine (Diener Electronic, UK). The top PDMS layer includes the microchannel, which was fabricated using a J826 Prime 3D Printer and molding process (Fig. 1C). The lower PDMS layer contains a small magnet (Figs. S2, S3), as shown in Fig. S21 (N52 high-grade neodymium magnet, 5 mm × 1.5 mm × 1 mm), which was embedded within the PDMS to align precisely beneath the electrodes’ position when plasma bonding. Sodium hydroxide can dissolve the gallium oxide layer on the liquid metal drop and ensure a stable and repeatable droplet motion during actuation, which is essential for reliable pump cycling over extended operation (Fig. 2H)^54,55^. To prevent cross-contamination between the working fluid and the NaOH surrounding the liquid metal droplet, we have integrated a physical separator that isolates these fluids. Specifically, two air bubbles are injected inside the microchannel on either side of the NaOH to serve as barriers, effectively blocking any direct contact or mixing between the NaOH and the pumping fluid (Fig. S17). The length of the injected air bubble was controlled by calculating the bubble volume within the channel for different channel heights.

The active section of the LIMA pump is extremely compact, as shown in Fig. S21 (0.086 cm^3^). Following common practice^31,34,61^, we did not include the external power supply and control system in the volume as they are not physically embedded into the device and can vary based on the requirements and implementation. Moreover, in order to make the fabrication process of the LIMA more accessible, we have demonstrated in Fig. S21 integrating the LIMA pump into a small tube and its capability to generate reciprocal pumping (Fig. S21A, Supplementary Movie 7).

### Performance evaluation

In the pressure characterization of the LIMA pump, a pressure sensor (SSCDRRN060MDAA5, Honeywell, UK) was connected to the pump outlet in a closed system. This configuration allowed us to capture the peak pressure generated during operation. An example of the pressure generated by the LIMA pump over time, showcasing the ramp-up behavior of the pump, is demonstrated in Fig. 2D.

For flowrate characterization a raindrop sensor module (AZ, Deutschland) was employed, and the data was recorded through a data acquisition system (USB-6211, National Instruments). Data was recorded through a data acquisition system (USB-6211, National Instruments). We investigated the effect of different variables, including the current applied (2 Amps to 8 Amps), length of the droplet (0.5 cm to 1.5 cm) and channel height (0.1 mm to 1 mm), on the value of the pressure and flow rate generated. The LIMA pump’s fluidic power generation also depends on the frequency of the alternating voltage applied. Here, we fix the pump amplitude using two infrared sensors, defining the endpoint of movement of the liquid metal droplet. Frequency of pumping was a passive emergent variable, dependent on experimental parameters including droplet size and sensor position. In Fig. S20, we also demonstrate that by integrating sensing electrodes, we can replace the infrared sensors and achieve a simpler system. This sensor can be utilized with an impulse latching relay to enable a self-oscillating pump (Fig. S22).

The LIMA pump was tested over extended periods of continuous operation (Fig. S14) without demonstrating any significant change in its operating mechanism. Displacement generated by the pump was quantified by tracking a floating marker using a laser displacement sensor. Over long durations, the marker exhibited slight tilting and positional drift, resulting in a small baseline shift in the recorded data. This effect is attributed to the measurement setup rather than to changes in actuator performance and is therefore not considered a failure mode of the LIMA pump. Importantly, LIMA operates at driving voltages below 1 V, which are below the electrolysis threshold, thereby avoiding the degradation associated with electrochemical reactions commonly reported in prior liquid-metal actuators^62–64^.

We also investigated the effect of using four passive check valves (AirLogic, US) on the LIMA pump’s performance. The check valves were connected in pairs on both sides of the pump outlets. The maximum block pressure and flow rate of the system are demonstrated in Fig. S16. To measure cracking pressure (Fig. S15), a pressure sensor is connected between the pair of check valves on one side of the pump, with the pump outlet open. To compare the performance of the LIMA pump with other soft and commercial pumps, we present the performance data in Fig. 1F as an Ashby plot; additional information can be found in Table S1^31,43,61,65–69^.

### Compact and standalone LIMA pump

To create a compact system, four check valves (AirLogic, US) were integrated on the sides of the microchannel to enable directional pumping. The magnetohydrodynamics actuation mechanism allows the pump to be powered by a single AA battery. The oscillation frequency of the liquid metal was controlled using a small electronic control circuit, with the potential for further miniaturization. A power switch was installed to control the on/off operation. The electronic control circuit utilizes an Arduino Nicla Sense ME microcontroller for logic operations, powered by a single lithium coin cell battery and interfaced with an H-bridge circuit. The H-bridge serves as the switching stage for a secondary, independent power source, a single 1.5 V AA alkaline battery, connected directly to the pump. The Arduino directs the current from the AA battery through the pump, enabling unidirectional flow. Future work will focus on implementing fully integrated flat valves and simplified driving electronics, for example, by replacing the Arduino and H-bridge with a simple two-transistor oscillator, to further reduce system complexity and reliance on discrete components.

### Butterfly robot and wearable wristband

The fabrication of the body of the butterfly robot (Acetate Sheets, HaberCrafts Ltd, UK) was accomplished using a computer-controlled cutting machine (Cricut Maker, Cricut, USA). In order to actuate the butterfly’s wings, we passed shape memory alloys (SMAs) (Biometal helix, Toki corporation, Japan) through the small holes in the wings. The wearable wristband is fabricated using a strap with elements 3D printed (Flashforge, China) using thermoplastic polyurethane (TPU). In order to actuate the wristband, we used SMAs (Biometal helix, Toki corporation, Japan) attached to the TPU 3D printed parts, such that actuation of the SMAs causes contraction. The flapping motion and the contraction of the wristband were induced through a code pattern generated by the LIMA pump. The power source for the SMAs (0.9 V) was independent of the power supply for the pump.

### Arthropod-inspired leg actuators

The manufacturing procedure for the spider-like legs included the fabrication of a slender sleeve from Ecoflex 00-10 silicone (Smooth-On Inc.), that was cast a 3D-printed mold. Within this sleeve, two tubes (Platinum cured silicone tubes, PVC Pipe World LTD, UK) with an outer diameter of 3 mm and an inner diameter of 1 mm were inserted, leaving a small gap between their respective ends. The sleeve-tube connections and one end of a pipe were sealed using silicone adhesive (Sil-Poxy, Smooth-On Inc.).

### 3D printed display bracelet

The DNA-inspired bracelet was designed using SolidWorks software and 3D printed (J826 prime 3D Printer, Stratasys) using Vero clear, Agilus clear and SUP706B materials.

### Haptic actuators

The first haptic actuator was fabricated using Ecoflex 00–30 (Smooth-On Inc.) with dimensions of 2 cm × 3 cm × 0.5 cm, cast in a 3D-printed mold. The second haptic actuator used in the tactile device experiment was fabricated using Ecoflex 00–10 (Smooth-On Inc.) with dimensions of 2 cm × 1 cm × 0.5 cm, cast in a 3D-printed mold. Using a strap, the actuator was fixed on the finger. For the chemical energy delivery, the acid and alkali chemicals used were 0.9 M sodium bicarbonate (Sigma-Aldrich, UK) in water and 1.3 M citric acid (Sigma-Aldrich, UK) in water, respectively. To show the differentiation between the two solutions, we added liquid color dyes (Honeyberry International LLP, UK).

### Finite element analysis

The electric potential (V) and current density (A/m^2^) of the LIMA pump at the three main positions within the channel (far left, center, and far right) using COMSOL Multiphysics 5.6 were simulated (Fig. S9).

## Supplementary information

Supplementary Information

Description of Additional Supplementary Files

Supplementary Movie 1

Supplementary Movie 2

Supplementary Movie 3

Supplementary Movie 4

Supplementary Movie 5

Supplementary Movie 6

Supplementary Movie 7

Supplementary Movie 8

Supplementary Movie 9

Transparent Peer Review file

## Data Availability

Data supporting the findings of this study are openly available in the University of Bristol Research Data Repository at: 10.5523/bris.hc5lgrn65qm01yfcg7gfr5ogl.
