# Supplementary material for: A flexible liquid metal magnetohydrodynamic pump for soft robotic systems
Source: Nat Commun. 2026 May 12;17:6345. doi: 10.1038/s41467-026-72798-7 (PMC13376768; doi:10.1038/s41467-026-72798-7)
Supplement: Supplementary file 1 — Supplementary Information [file 41467_2026_72798_MOESM1_ESM.pdf]

Supplementary Information

A flexible liquid metal magnetohydrodynamic pump for soft robotic systems

S. Firouznia, C. Romero, C. Xu, L. Tan, A. Stinchcombe, M. Garrad, A. Conn, H. Philamore, J. Rossiter*

* Correspondence to: jonathan.rossiter@bristol.ac.uk

Contents **Page**

1. Supplementary Figures 2
2. Supplementary Tables 24

1 Supplementary Figures

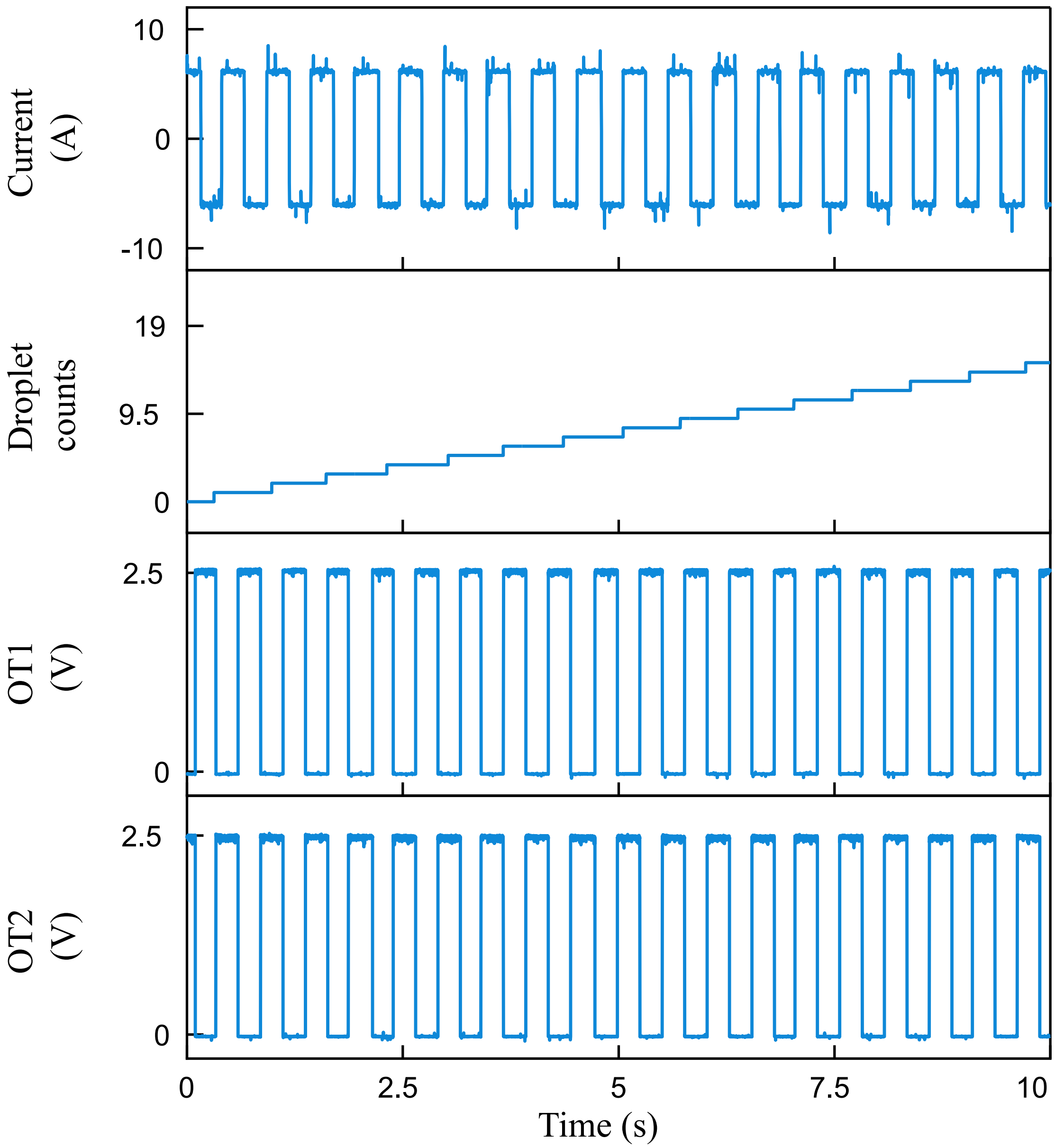

Fig. S1. A sample of data characterising the flow rate of the pump. The current through the liquid metal is measured in the system. The droplet count indicates the number of droplets detected by the sensor, corresponding to the droplets pumped by the device. Optical Transmission 1 (OT1) and Optical Transmission 2 (OT2) represent the voltages recorded by optical sensors positioned on the left and right sides of the electrode. These sensors precisely detect the placement of the liquid metal and enable dynamic voltage control based on its position.

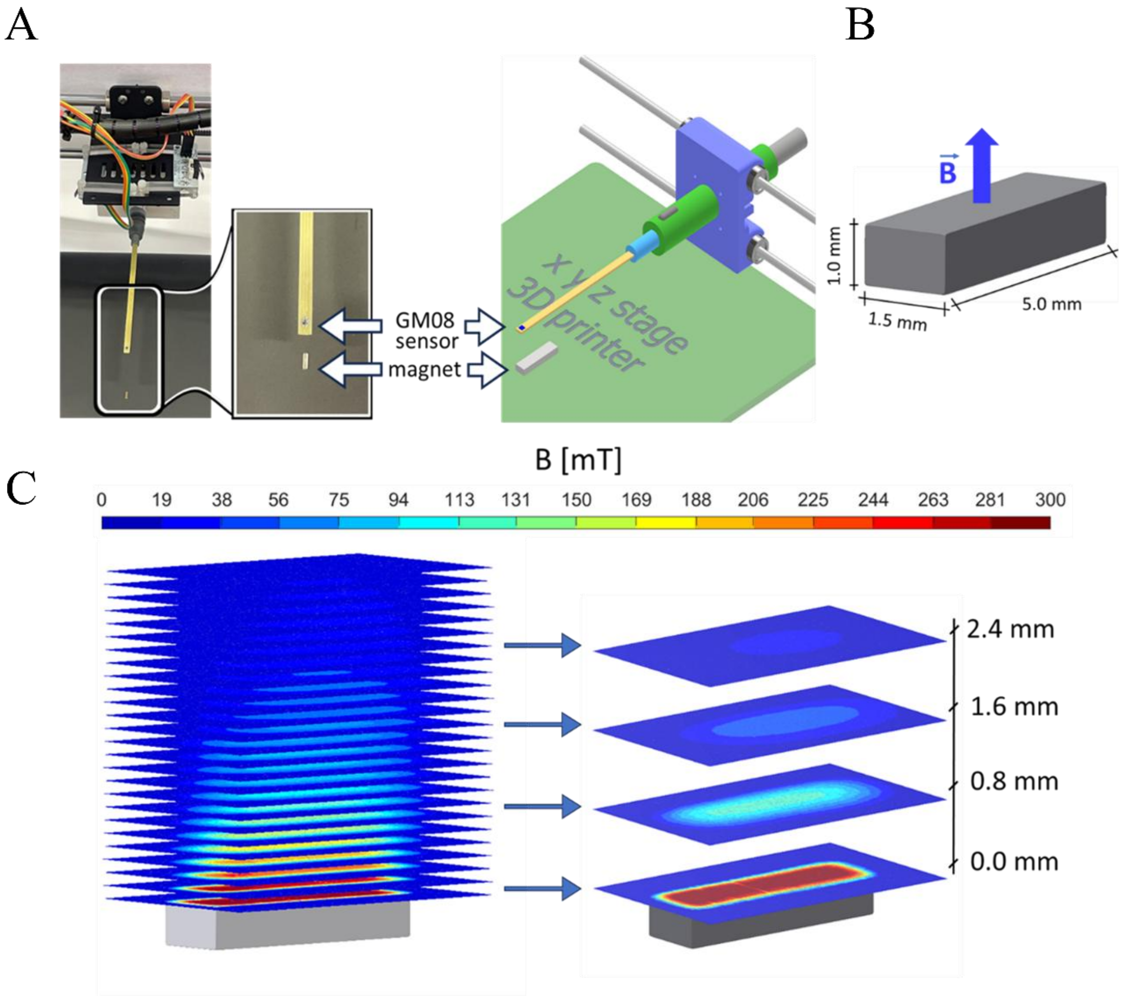

Fig. S2. Magnetic field characterization. A) GM08 gaussmeter probe mounted to the xyz stage of a commercial 3D printer (I3 Mega S, ANYCUBIC, CN). B) Magnet placed with the magnetic field facing upwards. C) 2D-profiles of the magnetic field intensity taken at different heights from the surface of the magnet. The magnetic field characterization was performed on a sample permanent magnet (1.0 mm × 1.5 mm × 5.0 mm, Magnosphere GmbH, GER). Magnetic field measurements were acquired using a precision gaussmeter (GM08 gaussmeter, Hirst Magnetic Instruments Ltd, UK). Readings of the normal component of the magnetic field were automatically collected at heights from 0.1 to 3.5 mm above the magnet's surface, forming 2D slices of magnetic field intensity (parallel to the plane of the magnet's surface).

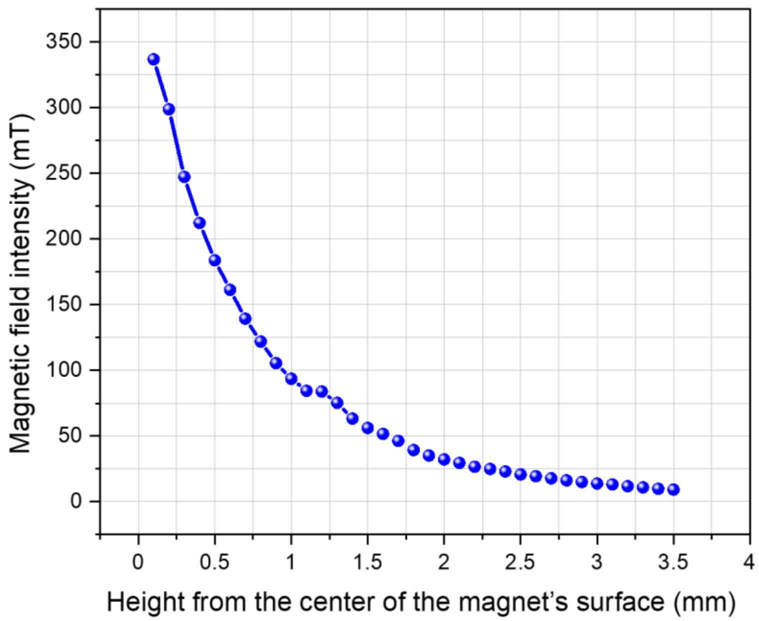

Fig. S3. Magnetic field and proximity: Magnetic field intensity taken at different heights from the center of the surface of the magnet. The magnetic field intensity recorded at the surface of the magnet (h = 0.1 mm) is 336.81 mT. At 2.0 mm from the surface the magnetic field decreases to 32.19 mT which is 9.56 % of the original value at the surface. At a further distance of 3.5 mm, the intensity reduces to 9.19 mT, equivalent to 2.73% of the magnetic field strength found at the magnet's surface.

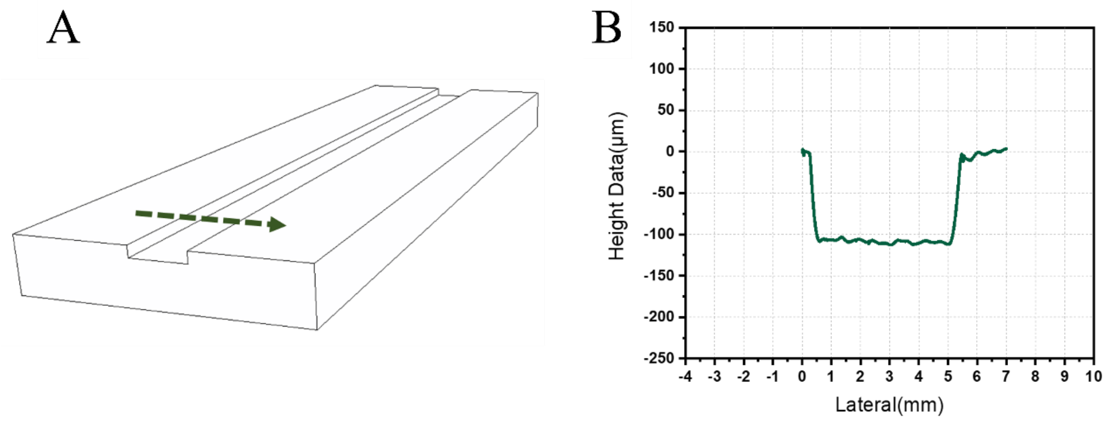

Fig. S4. Characterization of the PDMS microchannel. A) Sweeping path of the stylus profilometer probe, B) Cross-section profile of the PDMS microchannel. The size of the microchannels of the PDMS-based components of the LIMA pump were characterized using the stylus (contact) profilometry technique. The samples were placed in a stylus profilometer (Dektak XT, Bruker Ltd, UK) stage with the microchannel facing upwards. The measurements were performed by sweeping the stylus probe (2 μm radius tip) across the channel. The standard deviation from the data obtained is smaller than 3.4 μm.

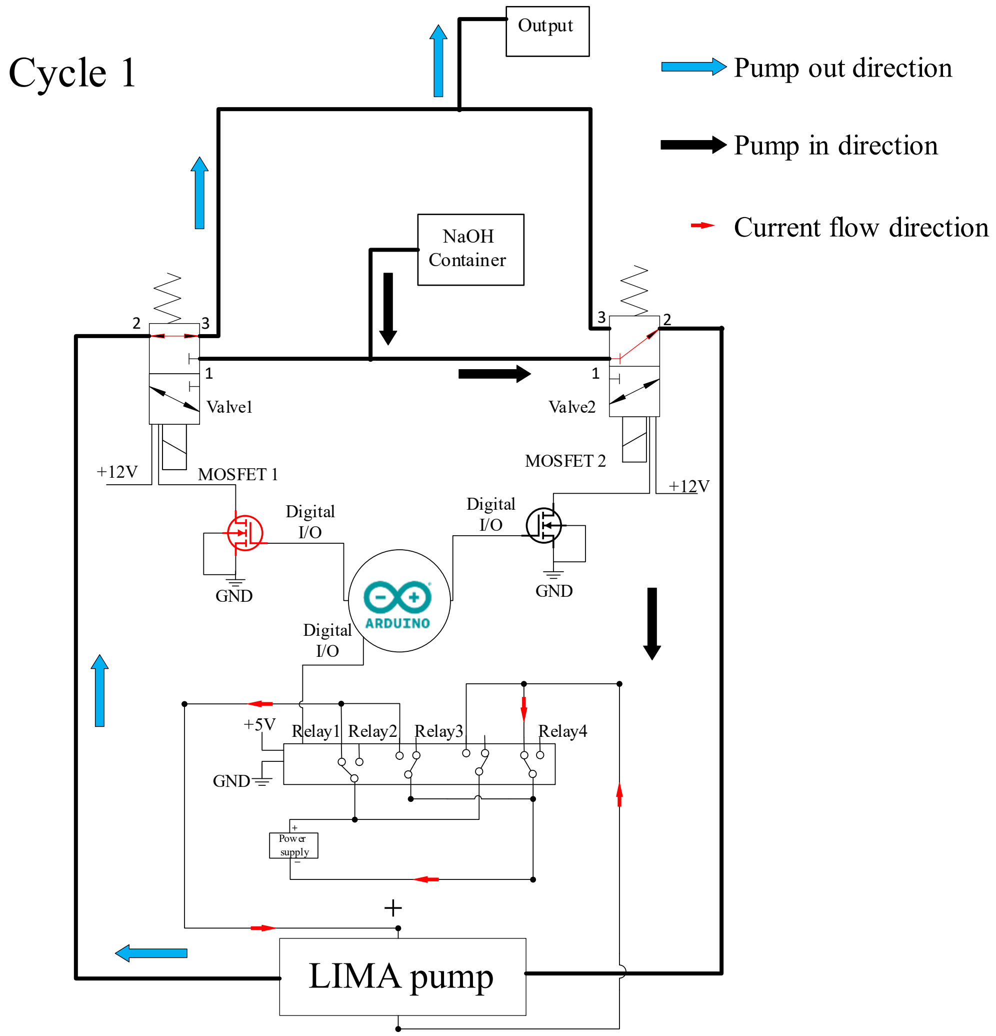

Fig. S5. Test setup electrical and flow diagram for cycle 1. The LIMA pump test system consists of the LIMA/MHD pump, two solenoid chemical valves (LVM095R, SMC), two logic level N-channel MOSFETs (PSMN1R8-30PL, NXP Semiconductors), four relays (SRD-05VDC-SL-C, Ningbo Songle Relay Co.,ltd.), Arduino UNO (SunFounder, China), output and input fluid containers, and a power supply. In cycle 1 (shown in Figure S5), the current from the positive terminal of the power supply passes through relay 1, flowing to the upper terminal of the MHD pump, then returns to the negative terminal of the power supply through relay 4. The current flow direction is shown by the small red arrows. When the electric current flows through the MHD pump, the liquid metal moves left because of the induced Lorentz force. Simultaneously, gates 1 and 2 are connected inside valve 2, driven by MOSFET1 (shown in red). The differential pressure generated makes the NaoH flow as shown by the blue arrows (pump outlet) and black arrows (pump inlet).

Cycle 2

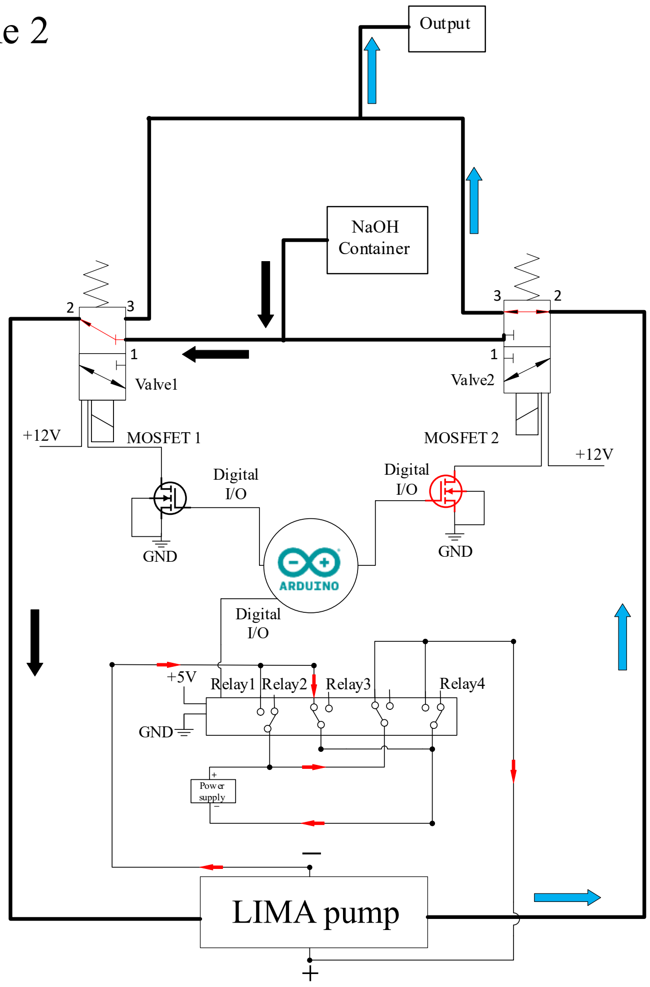

Fig. S6. Test setup electrical and flow diagram for cycle 2. In cycle 2 the current from the positive terminal of the power supply passes through relay 3, flowing to the lower terminal of the LIMA pump, then returns to the negative terminal of the power supply through relay 2, shown by small red arrows. In this stage the induced Lorentz force makes the liquid metal move right. Simultaneously, MOSFET2 drives valve 2 to connect gate 3 and gate 2 (shown in red). The generated pressure difference makes the NaOH flow in the direction shown by the black and blue arrows.

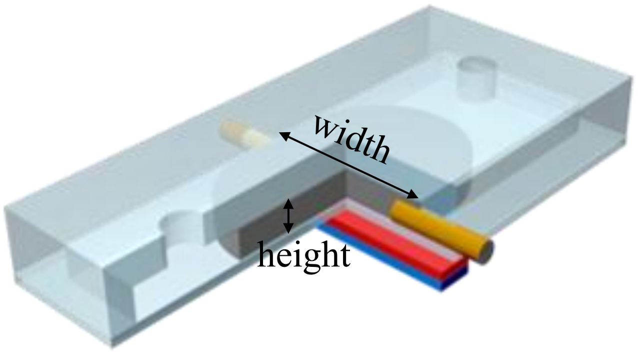

Fig. S7. Microfluidic channel characteristics.

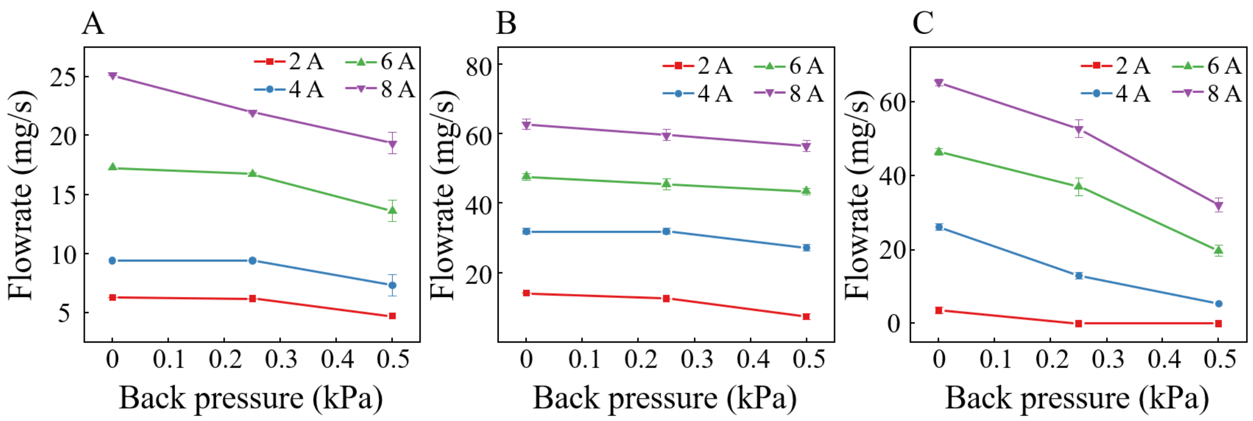

Fig. S8. The flow rate against different backpressures for different currents. A) Flow rate against backpressure for channel height 0.1 mm. B) channel height 0.3 mm. C) channel height 0.5 mm.

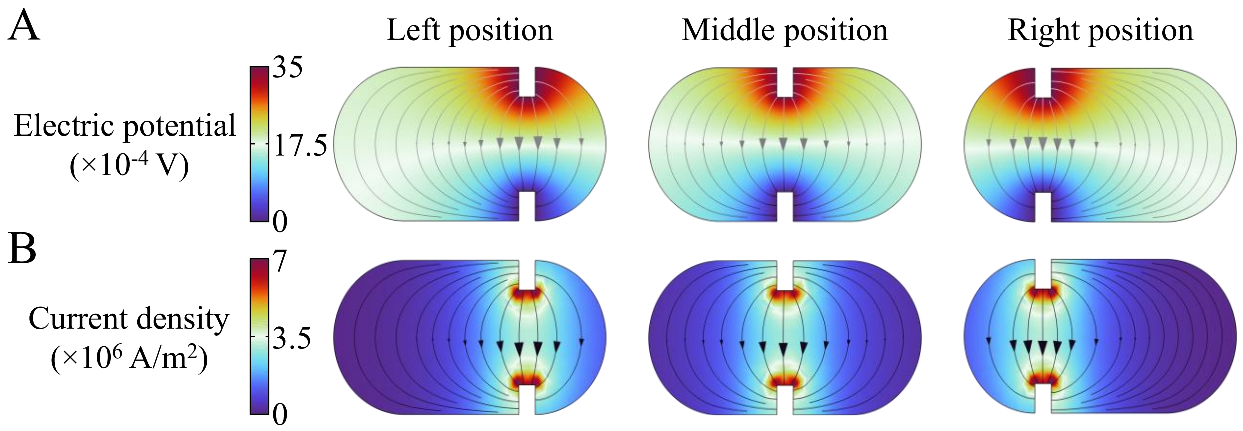

Fig. S9. Electrical finite element simulations (COMSOL) of the liquid metal droplet. A) Distribution of electric potential, B) Distribution of current density for three stages of the LIMA pump. In our experimental work, there is a limit to how closely we can measure the droplet's voltage in situ; therefore, to determine the additional resistance introduced by wiring, we simulated the droplet in the three main stages of the LIMA pump and calculated the modelled efficiency as 0.25 per cent.

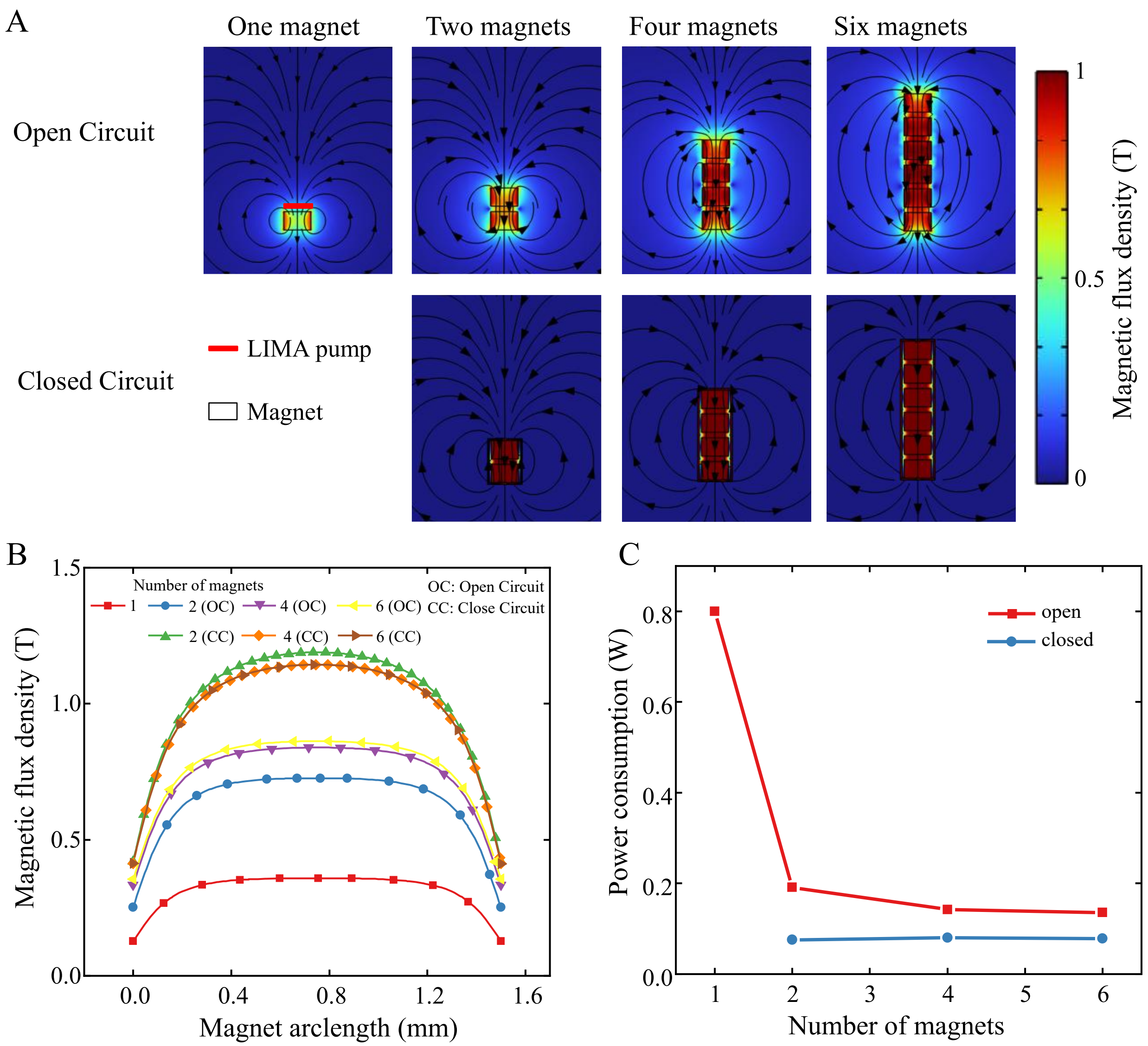

Fig. S10. Demonstrating the effect of magnetic stacking and magnetic circuits on the LIMA pump power consumption. A) COMSOL study on effect of number of magnets and magnetic encasement. The magnetic flux density increases significantly by adding an encasement. B) The magnetic flux density along a line 0.15 mm above the surface of the magnet (representing the middle of the microchannel). C) By increasing the magnetic flux density, the required current to achieve the same Lorentz force will be reduced, reducing power input to the LIMA pump. Input power decreases by up to 9 times using the magnetic circuit configuration with two magnets.

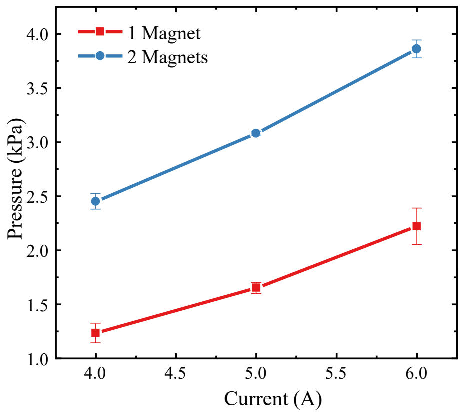

Fig. S11. Pressure against applied current for the LIMA pump (height 0.1 mm) with one and two magnets.

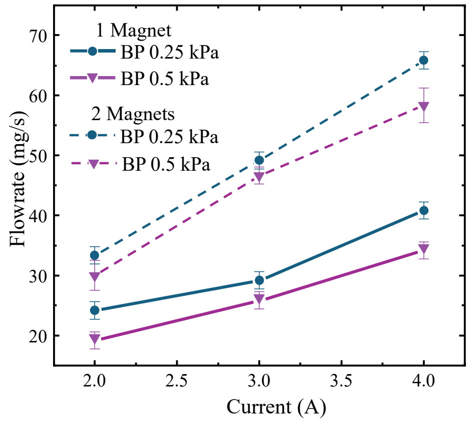

Fig. S12. Flow rate against the current with back pressure for the LIMA pump (height 0.3 mm) with one and two magnets. The efficiency of the open-magnet system is 0.02 per cent, which is improved significantly by closing the magnetic circuit, as demonstrated in Fig. S9.

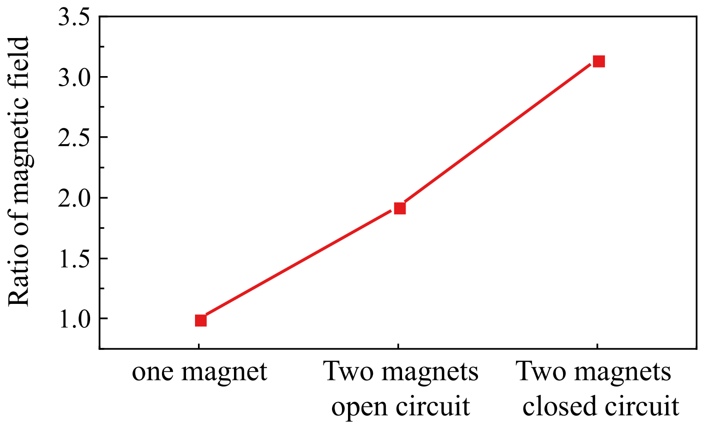

Fig. S13. Experimental results showing increase in magnetic field from one magnet to two magnets with an open circuit and two magnets with a closed circuit.

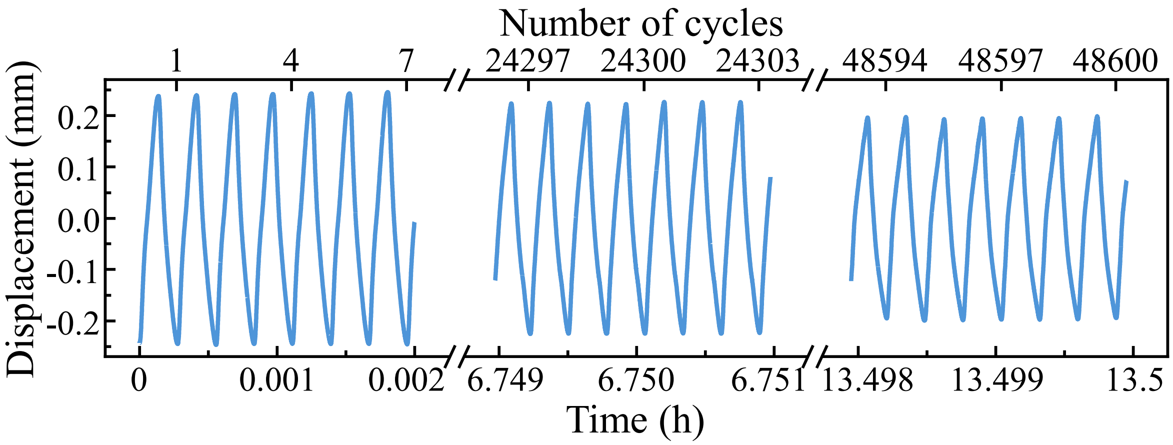

Fig. S14. Cyclic test for LIMA pump. The displacement induced from the cyclic actuation of the LIMA pump was operated for 13.5 hours at 1 Hz, corresponding to 48,600 cycles. The small change in the displacement is due to minimal oxidation of the liquid metal droplet over high operating cycles.

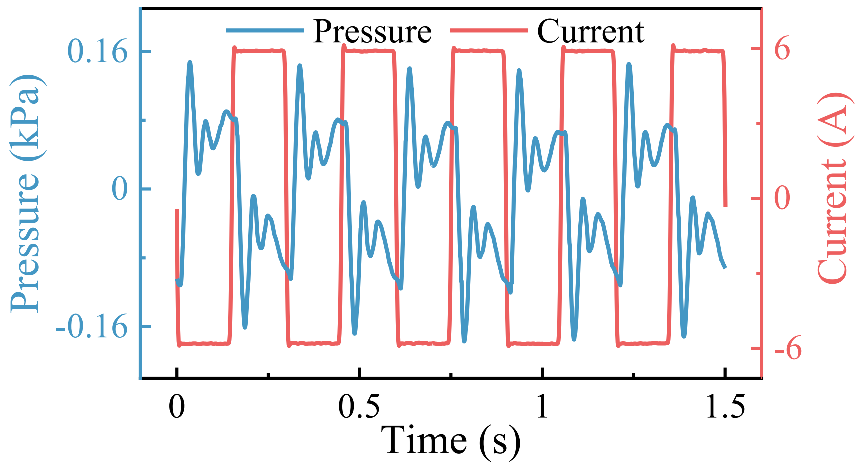

Fig. S15. Measured pressure and applied current to the LIMA pump. The pressure sensor was positioned between the two check valves on one side of the pump to measure the rectified pressure. The square-wave current drives cyclic actuation of the liquid metal droplet, while the resulting periodic pressure response demonstrates unidirectional pumping enabled by the check valves, with superimposed oscillations induced by check-valve dynamics and fluid inertia.

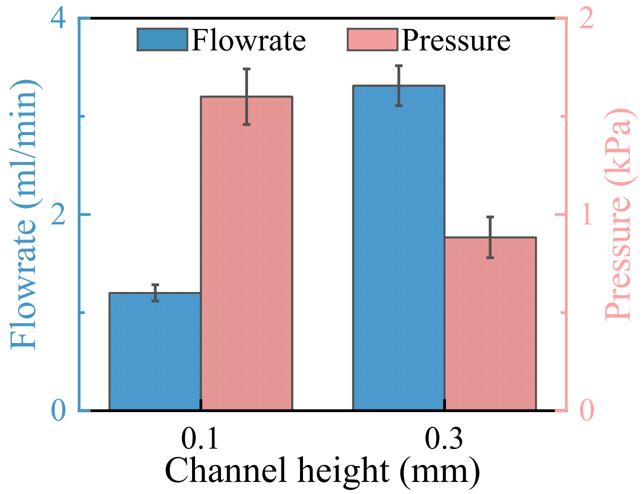

Fig. S16. The maximum measured block pressure and flow rate of the LIMA pump utilising four passive check valves for the samples with heights of 0.1 mm and 0.3 mm, with an applied current of 8 Amps.

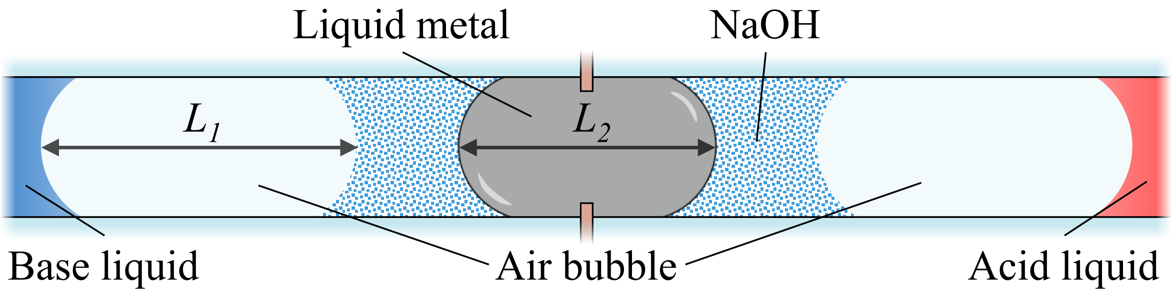

Fig. S17. Separation of two different liquids. The integrated air gap serves as a physical barrier, ensuring that pumped fluids remain separate and preventing cross-contamination. Both sides of the liquid metal droplet are coated with NaOH to prevent oxidation. Air gaps on the left and right isolate the pumping fluids. Moreover, the air bubble's length (L_1) exceeds the maximum stroke amplitude of liquid metal (L_2) to ensure reliable separation throughout the actuation cycle. No noticeable change in the pump's performance was observed due to the integration of air bubbles.

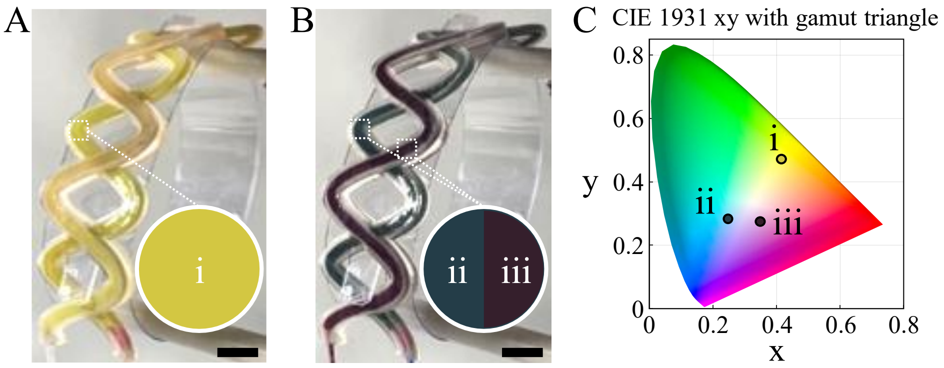

Fig S18. Color change of the bracelet. A) The color before fluid injection. B) Colors after fluid injection. C) The CIE 1931 colour gamut of the three colours of the bracelet. (i-iii) in (A-C) are matched. White balance normalisation was applied to images in (A) and (B). Scale bars are 5 mm.

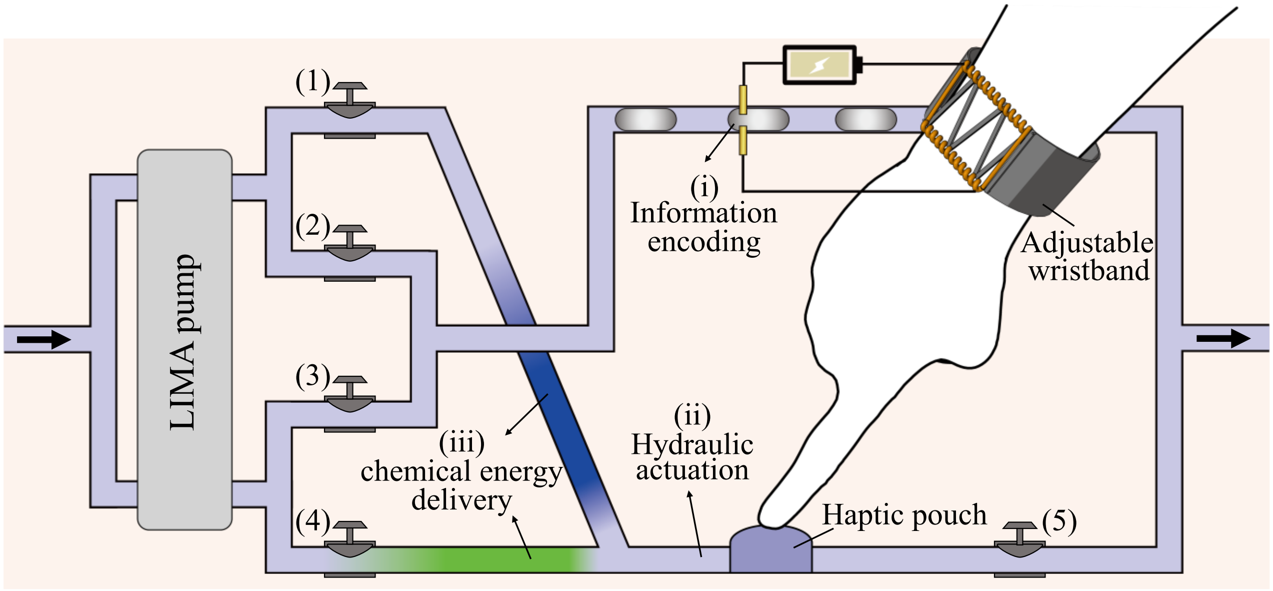

Fig. S19: Schematic diagram of the integrated haptic device demonstration using a single LIMA pump to deliver three distinct functional modes sequentially: (i) information encoding, (ii) hydraulic actuation, and (iii) chemical energy delivery. A coded liquid metal signal activates the wristband to apply circumferential force in the first mode. In the second mode, the user senses a gentle pressure on their finger via hydraulic actuation. In the third mode, a higher pressure is generated by gas production from the chemical reaction. This setup demonstrates the integration of three network-like modalities within a single system. Each of these modalities can be directly driven by a separate LIMA pump to avoid system complexity. In this demonstration, valves were used to manually control the timing of each mode to avoid sensory overlap. For the first mode, valves 1 and 4 are closed, and 2 and 3 are open, to generate the code pattern and then pump the code to activate the circuit of the SMA-driven wristband. For the next mode, the 2, 3, and 5 are closed, and 1 and 4 are open to actuate and pressurise the actuator on the finger by inflating it using the hydraulic actuation. For the last mode, mode 5 is first opened for the two chemicals to reach close to the mixing point, and valve 5 is closed for the third mode chemical reaction actuation.

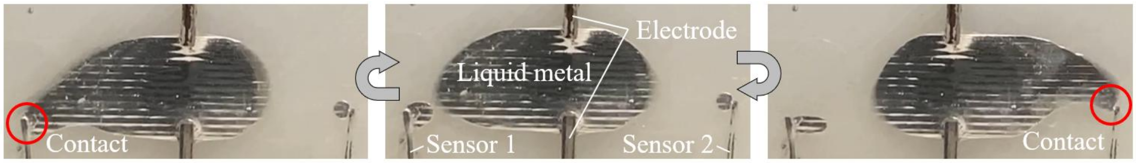

Fig. S20. Employing two sensor electrodes to detect the stroke endpoints of the LIMA pump. The droplet length and sensor wire placement determine the stroke amplitude.

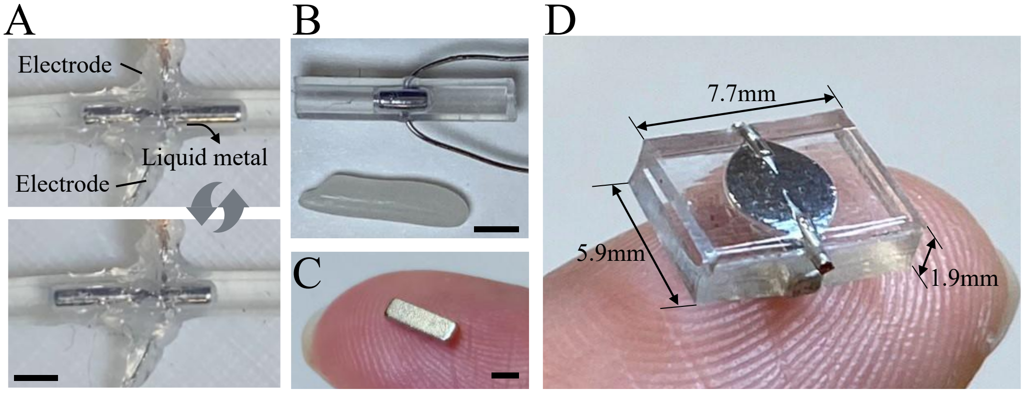

Fig. S21. Demonstrating the simplicity and compact design of the LIMA pump. A) Integrating the LIMA pump into a small tube (scale bar 2 mm) and demonstrating reciprocal pumping (Supplementary Movie 7). B) The tube pump shown next to a grain of rice (scale bar 2 mm). C) The magnet utilised in the LIMA pump (scale bar 2 mm). D) The LIMA pump used in evaluation tests.

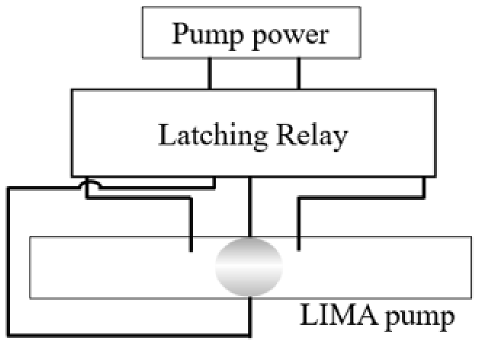

Fig. S22. A potential simplified circuit for creating a self-oscillating LIMA pump. By using latching relays and the two integrated sensing electrodes it is possible to further simply the system to avoid using any control system for generating the alternating voltage.

2 Supplementary Tables

Table S1. Comparison of pump performance with leading soft and commercial pumps

Mechanism	Ref.	Mass (gr)	Vol (cm^3)	Max power consumption (W)	Max pressure (kPa)	Max flowrate (ml/min)	Pressure/ size (GPa/m^3)	Flowrate/ size (kl/min m^3)
				Soft pumps				
MHD	This work	0.2	0.086	0.8	a) 3	4.2	34.8	49.3
					b) 1.6	3.3	18.6	38.4
EHD	*(31)*	1	1.17	0.17	14	6	12	5.13
EHD	*(65)*	1.2	10.05	0.71	80	45	7.96	4.5
MHD	*(43)*	30	22.7	0.17	8	320	0.35	14
DEA	*(61)*	13.5	15	3.3	12.5	39	0.83	2.6
Magnetic	*(66)*	12.8	11.6	4	0.001	134	0.0001	11.5
				Commercial pumps				
MGD 1000S	*(67)*	142	58.6	30	800	500	13.7	8.5
McMaster STPAC	*(68)*	15422	75500	1200	1034	42500	0.01	0.56
Gotec ESX-04	*(69)*	35	24.6	5	30	83.3	1.2	3.38

Note:
a) Pumps incorporated with solenoid valves.
b) Pumps incorporated with check valves.
Acronyms: MHD, EHD, and DEA represent magnetohydrodynamics, electrohydrodynamics, and dielectric elastomer actuators, respectively.
