# Supplementary material for: A flexible liquid metal magnetohydrodynamic pump for soft robotic systems
Source: Nat Commun. 2026 May 12;17:6345. doi: 10.1038/s41467-026-72798-7 (PMC13376768; doi:10.1038/s41467-026-72798-7)
Supplement: Supplementary file 2 — Description of Additional Supplementary Files [file 41467_2026_72798_MOESM2_ESM.pdf]

Description of Additional Supplementary Files

Supplementary Movie 1. Demonstration of LIMA pump flexibility

Supplementary Movie 2. Control capabilities of the LIMA pump

Supplementary Movie 3. The butterfly wing actuation is controlled by the code produced by the LIMA pump

Supplementary Movie 4. Actuating a soft arthropod-inspired leg by inflating the soft joint

Supplementary Movie 5. Demonstration of bidirectionality of LIMA pump

Supplementary Movie 6. Demonstration of the fluidic capabilities of LIMA pump

Supplementary Movie 7. Demonstrating the simple design of the LIMA pump.

Supplementary Movie 8. Integration of information encoding, hydraulic, and chemical actuation in a unified soft wearable

Supplementary Movie 9. A compact LIMA pump system
