# Supplementary material for: A flexible liquid metal magnetohydrodynamic pump for soft robotic systems
Source: Nat Commun. 2026 May 12;17:6345. doi: 10.1038/s41467-026-72798-7 (PMC13376768; doi:10.1038/s41467-026-72798-7)
Supplement: Supplementary file 12 — Transparent Peer Review file [file 41467_2026_72798_MOESM12_ESM.pdf]

nature portfolio

Peer Review File

A flexible liquid metal magnetohydrodynamic pump for soft robotic systems

Corresponding Author: Professor Jonathan Rossiter

This file contains all reviewer reports in order by version, followed by all author rebuttals in order by version.

Version 1:

Reviewer comments:

Reviewer #1

(Remarks to the Author)
This manuscript presents an embeddable liquid metal soft magnetohydrodynamic displacement pump, offering a new solution for fluid actuation in soft robotics. Lightweight and compact characteristics are achieved through optimization of microchannel geometry, magnetic field configuration, and fluid composition. The manuscript highlights its versatility like powering a macroscale soft actuator, transferring chemical fluids and generating diverse code patterns for simultaneous information and energy transfer. In general, the manuscript is well organized and written and the conclusions are consistent and supported by the results. This work could be considered for acceptance after the authors address the following comments.

1. The authors conducted a 10,000 second cycling test on LIMA, and the driving performance appears to remain largely unchanged. Could the authors clarify what the typical lifetime of LIMA is in terms of the number of operational cycles? Additionally, what are the primary failure modes of the device?
2. In the experiment mixing sodium bicarbonate and citric acid (Fig. 4C), how did the authors prevent sodium bicarbonate from coming into contact and reacting with NaOH, and likewise prevent acid from reacting with NaOH?
3. In the performance characterization section, the reviewer agrees with the decision not to include the external power supply in the reported device volume. However, in the comparison studies (Refs. 31, 34, 42), either no valves were used or only passive check valves were employed. In this work, LIMA relies on solenoid valves for a continuous one-way pumping action, which effectively avoids the energy loss required to overcome the breaking pressure of passive check valves. This gives LIMA a distinct advantage and makes the comparison with other studies not directly comparable. Therefore, the reviewer suggests that the measurements of maximum flowrate and maximum blocked pressure should be obtained under settings using four passive check valves, or alternatively, the volume of the solenoid valves should be included in the volume of LIMA.
4. Minor issues:
- The data in Fig. 2(H) appear discontinuous and distorted.
- The signal traces in Fig. 3(D) seem to be hand-drawn; actual recorded signals should be used instead.
- Ref. 45 and 46 are missing.
- The reference numbers cited in Table S1 do not correspond to the entries in the references.

Reviewer #2

(Remarks to the Author)
This manuscript, titled “A flexible liquid metal magnetohydrodynamic pump for soft robotic systems,” presents the design, characterization, and validation of a soft mechanical pump actuated by a movable liquid metal droplet. The actuation mechanism is based on Lorentz forces generated when electric current passes through the liquid metal. The pump is soft except for a small embedded magnet beneath the droplet. Three demonstrations are included to illustrate the potential applications of the pump: driving a macroscale soft actuator, converting chemical energy to mechanical energy, and generating varied code patterns for information transfer. The reviewer has the following comments and questions.

1. Claims of advantages over existing technologies are not convincingly supported.

The liquid-metal-based pump is an interesting concept. However, the demonstrations do not clearly establish the

advantages or practical benefits over existing pumps.

In the Abstract, two main limitations of existing systems are described as “portability” and “system complexity.” It is not evident that the proposed pump effectively addresses either:

(a) Portability:
The pump is largely soft and operates at a lower voltage, which could theoretically improve portability. However, the manuscript does not provide characterization, comparison, or evidence that demonstrates enhanced portability relative to existing devices.

(b) System complexity:
The main figures and text present the pump as a simple system. However, details in the Supplementary Information suggest otherwise. For example, the setup in Fig. S19 for the Fig. 5 experiment includes multiple valves and branches. This does not account for off-board peripherals such as the power supply or fluid sources. As presented, there is no direct evidence that the overall system is less complex than existing alternatives.

In addition, several demonstrations appear overstated relative to the preliminary nature of the results:

(a) The spider robot shown in Fig. 1A is not directly supported by the experiments in the manuscript. It is unclear how the proposed pump could feasibly enable such a system. Given the available volume in that hypothetical robot, more established rigid pumps could be used. The figure raises questions about relevance and practicality. The reference to “chromatophores” is also unclear and not integrated elsewhere in the manuscript.

(b) Referring to the bracelet as a “display” may overstate its function.

(c) The term “information encoding” is used to describe control of an LED or a shape memory alloy via droplet distribution. While this may be technically defensible, it is not clear that the demonstrations meaningfully qualify as information encoding. The butterfly example in Fig. 3C does not appear to add substantive insight; its relevance to pump design or function is not explained.

2. Additional clarification is needed for certain experiments.

For the experiment in Fig. 4A, the bidirectional operation of the pump is not clearly explained. How is flow direction controlled?

For the experiment in Fig. 5, the control method for the three operation modes (manual vs. automatic) should be described. The text mentions “participants,” but only one individual is shown. Details such as the number of participants, experimental procedure, and number of trials per participant should be provided.

3. Limitations of the Fig. 5 demonstration should be acknowledged.

The third operational mode in Fig. 5 appears to be single-use only. This limitation should be clearly stated in the manuscript.

Version 2:

Reviewer comments:

Reviewer #1

(Remarks to the Author)
The authors have alleviated my concerns and corrected the identified errors. The manuscript is now in a much stronger position and can be recommended for publication.

Reviewer #2

(Remarks to the Author)
The reviewer thanks the authors for making substantial efforts to address the concerns raised in the previous round of peer review. In particular, the removal of the spider robot figure and the addition of a physical, untethered prototype in the new Fig. 3 significantly strengths the manuscript. And the newly added details and explanations about the experiments are also appreciated. While now the manuscript is notably more solid, some of the underlying issues regarding the preliminary nature of the demonstrations remain. The concepts of this work is very clear, but the practical execution is still mostly rudimental and proof-of-concept.

1. Portability and complexity

The newly added standalone pump system (Fig. 3) demonstrates that the pump can operate in an untethered mode. This addition addresses the portability concern. However, the claim of “low complexity” remains. To achieve this portable operation, the system requires four one-way valves, a custom H-bridge, an Arduino Nicla Sense ME microcontroller, a

lithium coin cell, and an AA battery. Even though the pump itself is structurally simple, the peripheral hardware required to make it function is not less complex than what is required for existing miniature pumps.

2. Claims about demonstrations

Defining the experiment as “spatiotemporal signal encoding” and discussing it in the context of “computational systems” and “information processing” [the newly added last paragraph of Introduction] still feel somewhat inflated considering the simple switch-closing mechanic demonstrated.

3. The haptic device (Fig. 6)

The reviewer thanks the authors for the clarification of the number of participants and the experiment procedure. But the newly added explanations do not change the preliminary nature of this specific demonstration. An experiment with one participant like this is a good proof-of-concept, but not a validated human-robot interaction study.

Open Access This Peer Review File is licensed under a Creative Commons Attribution 4.0 International License, which permits use, sharing, adaptation, distribution and reproduction in any medium or format, as long as you give appropriate credit to the original author(s) and the source, provide a link to the Creative Commons license, and indicate if changes were made.
In cases where reviewers are anonymous, credit should be given to 'Anonymous Referee' and the source.
The images or other third party material in this Peer Review File are included in the article’s Creative Commons license, unless indicated otherwise in a credit line to the material. If material is not included in the article’s Creative Commons license and your intended use is not permitted by statutory regulation or exceeds the permitted use, you will need to obtain permission directly from the copyright holder.
To view a copy of this license, visit https://creativecommons.org/licenses/by/4.0/

Responses to reviewers' comments

We very much appreciate the efforts and strong positive evaluations of the editor and reviewers of our paper. We have revised the manuscript based on the constructive comments of the reviewers. The comments from referees are reprinted in *blue italic*, and our responses are marked in black. Changes made to the manuscript are marked with red.

Here, we summarize the changes we made to the manuscript in response to reviewers' comments:

1. More detailed explanation regarding the effect of bidirectional pumping was added to the manuscript.
2. We have clarified our work by improving Figure 1 and removing the prior conceptual spider diagram.
3. We have clarified the limitations of the chemical energy-driven actuation.
4. We revised the manuscript to use more precise wording by describing the bracelet as a soft fluidic bracelet for visualization and added an additional figure showing the CIE 1931 colour gamut to clarify the bracelet's colour change.
5. We have clarified the method used for avoiding cross-contamination of working fluids, and a new diagram (Fig S17) and description were added to the manuscript and supplementary materials.
6. New experiments demonstrating the portability and compactness of the LIMA pump are shown in Figure 1C, 3, and Supplementary Video 9.
7. New experiments to demonstrate the operation of the LIMA pump using passive check valves are shown in Figure 1F and Fig.S15, S16.
8. New experiments showing the reliability of the LIMA pump over a longer period (13.5 hours) and updated the manuscript with new Fig.S14 and descriptions.

We have addressed the specific comments of all reviewers below:

REVIEWER COMMENTS

Reviewer #1:

This manuscript presents an embeddable liquid metal soft magnetohydrodynamic displacement pump, offering a new solution for fluid actuation in soft robotics. Lightweight and compact characteristics are achieved through optimization of microchannel geometry, magnetic field configuration, and fluid composition. The manuscript highlights its versatility like powering a macroscale soft actuator, transferring chemical fluids and generating diverse code patterns for simultaneous information and energy transfer. In general, the manuscript is well organized and written and the conclusions are consistent and supported by the results. This work could be considered for acceptance after the authors address the following comments.

1. The authors conducted a 10,000 second cycling test on LIMA, and the driving performance appears to remain largely unchanged. Could the authors clarify what the typical lifetime of LIMA is in terms of the number of operational cycles? Additionally, what are the primary failure modes of the device?

Response:

We appreciate the reviewer's observation. We have conducted new experiments to address this point. The LIMA pump was tested for 48,600 s (≈14 h) at 1 Hz, corresponding to 48,600 cycles. No significant degradation in performance was observed over the test duration, as shown in Fig.S14. The minor variation in sweep magnitude is attributed to the slight oxidation of the liquid metal. However, because no electrolysis and electrochemical reactions are involved in our work, we can demonstrate substantially longer operational lifetimes compared to prior liquid metal actuators (62-64). We have made the following changes to the manuscript and supplementary information to clarify the points raised.

Revision to the manuscript:

Page 8: In addition, the LIMA pump operated continuously for 48,600 s (13.5 h) at 1 Hz, accumulating 48,600 cycles. Over this extended duration, a minor change in actuation amplitude was observed, attributed to minimal oxidation of the liquid metal droplet, as shown in Fig. S14.

Revision to materials and methods:

Page 17: The LIMA pump was tested over extended periods of continuous operation (Fig. S14) without demonstrating any significant change in its operating mechanism. Displacement generated by the pump was quantified by tracking a floating marker using a laser displacement sensor. Over long durations, the marker exhibited slight tilting and positional drift, resulting in a small baseline shift in the recorded data. This effect is attributed to the measurement setup rather than to changes in actuator performance and is therefore not

considered a failure mode of the LIMA pump. Importantly, LIMA operates at driving voltages below 1 V, which are below the electrolysis threshold, thereby avoiding the degradation associated with electrochemical reactions commonly reported in prior liquid-metal actuators (62-64).

References:

(62) Liao, J., Majidi, C. and Sitti, M., 2024. Liquid metal actuators: a comparative analysis of surface tension controlled actuation. Advanced Materials, 36(1), p.2300560.

(63) Liao, J. and Majidi, C., 2022. Muscle - inspired linear actuators by electrochemical oxidation of liquid metal bridges. Advanced Science, 9(26), p.2201963.

(64) Tang, S.Y., Khoshmanesh, K., Sivan, V., Petersen, P., O'Mullane, A.P., Abbott, D., Mitchell, A. and Kalantar-Zadeh, K., 2014. Liquid metal enabled pump. Proceedings of the National Academy of Sciences, 111(9), pp.3304-3309.

Revision to the SI:

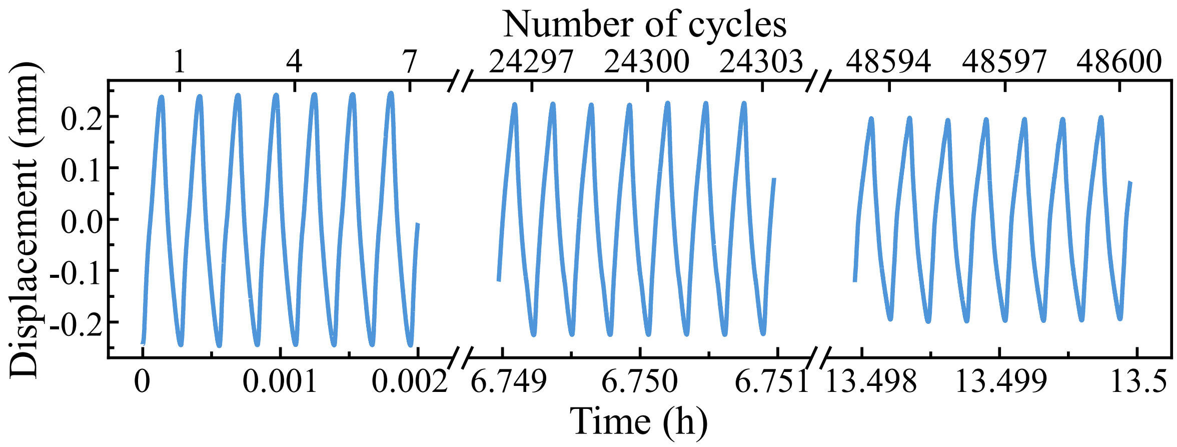

Fig. S14. Cyclic test for LIMA pump. The displacement induced from the cyclic actuation of the LIMA pump was operated for 13.5 hours at 1 Hz, corresponding to 48,600 cycles. The small change in the displacement is due to minimal oxidation of the liquid metal droplet over high operating cycles.

2. In the experiment mixing sodium bicarbonate and citric acid (Fig. 4C), how did the authors prevent sodium bicarbonate from coming into contact and reacting with NaOH, and likewise prevent acid from reacting with NaOH?

Response:

We appreciate the reviewer's insight. In our system, to physically isolate the pumping medium from the LM and the NaOH surrounding the droplet to prevent oxidation, we inserted air gaps on both sides of the NaOH barrier, as shown in Figure S21. The length of the air bubbles was set to exceed the liquid metal stroke length to ensure reliable fluid separation throughout the actuation. By integrating the air gaps, no noticeable change in the pumping performance was observed. To clarify, we have added the following changes to the manuscript.

Revision to the manuscript:

Page 12. Next, we demonstrated the unique capability of the LIMA pump to simultaneously pump different fluids, enabled by its differential outlet system and integrated air gap (see Fig. S17).

Revision to Materials and Methods:

Page 15. To prevent cross-contamination between the working fluid and the NaOH surrounding the liquid metal droplet, we have integrated a physical separator that isolates these fluids. Specifically, two air bubbles are injected inside the microchannel on either side of the NaOH to serve as barriers, effectively blocking any direct contact or mixing between the NaOH and the pumping fluid (Fig. S17). The length of the injected air bubble was controlled by calculating the bubble volume within the channel for different channel heights.

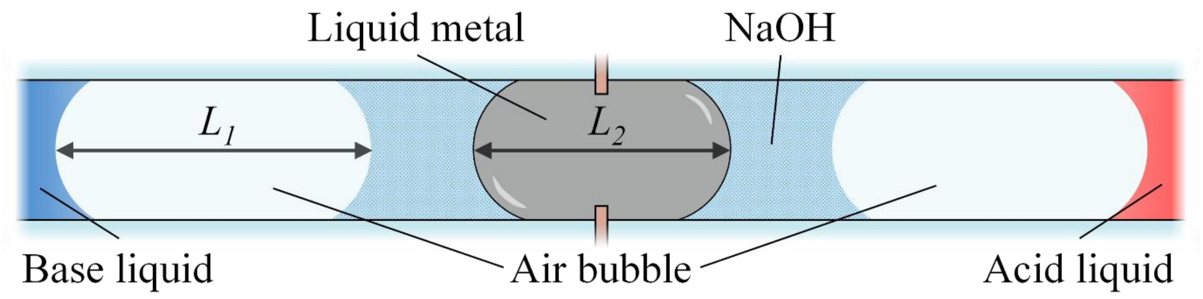

Fig S17. Separation of two different liquids. The integrated air gap serves as a physical barrier, ensuring that pumped fluids remain separate and preventing cross-contamination. Both sides of the liquid metal droplet are coated with NaOH to prevent oxidation. Air gaps on the left and right isolate the pumping fluids. Moreover, the air bubble's length (L_1) exceeds the maximum stroke amplitude of liquid metal (L_2) to ensure reliable separation throughout the actuation cycle. No noticeable change in the pump's performance was observed due to the integration of air bubbles.

3. In the performance characterization section, the reviewer agrees with the decision not to include the external power supply in the reported device volume. However, in the comparison studies (Refs. 31, 34, 42), either no valves were used or only passive check valves were employed. In this work, LIMA relies on solenoid valves for a continuous one-way pumping action, which effectively avoids the energy loss required to overcome the breaking pressure of passive check valves. This gives LIMA a distinct advantage and makes the comparison with other studies not directly comparable. Therefore, the reviewer suggests that the measurements of maximum flowrate and maximum blocked pressure should be obtained under settings using four passive check valves, or alternatively, the volume of the solenoid valves should be included in the volume of LIMA.

Response:

We thank the reviewer for their comment. We have conducted new experiments to achieve the highest flow rate and pressure using the LIMA pump with 4 check valves. As expected, we lose some pressure and flow rate when using check valves instead of solenoid valves. Nevertheless, as demonstrated by the Asby plot in Figure 1, we still lead in specific pressure and specific flow rate compared to prior work. Furthermore, since cracking pressure and stiction may occur during rapid flow direction changes, we will investigate this further in future work to develop check valves with lower cracking pressure and to avoid stiction at high frequencies. Please find the changes made to the manuscript and supplementary information below.

Revision to the manuscript:

Abstract: LIMA is lightweight (0.2 g) and compact (0.086 cm^3), delivering specific pressure (18.6 -34.88 GPa m^{-3}) and specific flow rate (38.4 - 49.29 kl min^{-1} m^{-3}), surpassing existing soft and conventional pumps, and operating at low voltage (<1 V).

Page 2: Figure 1.F

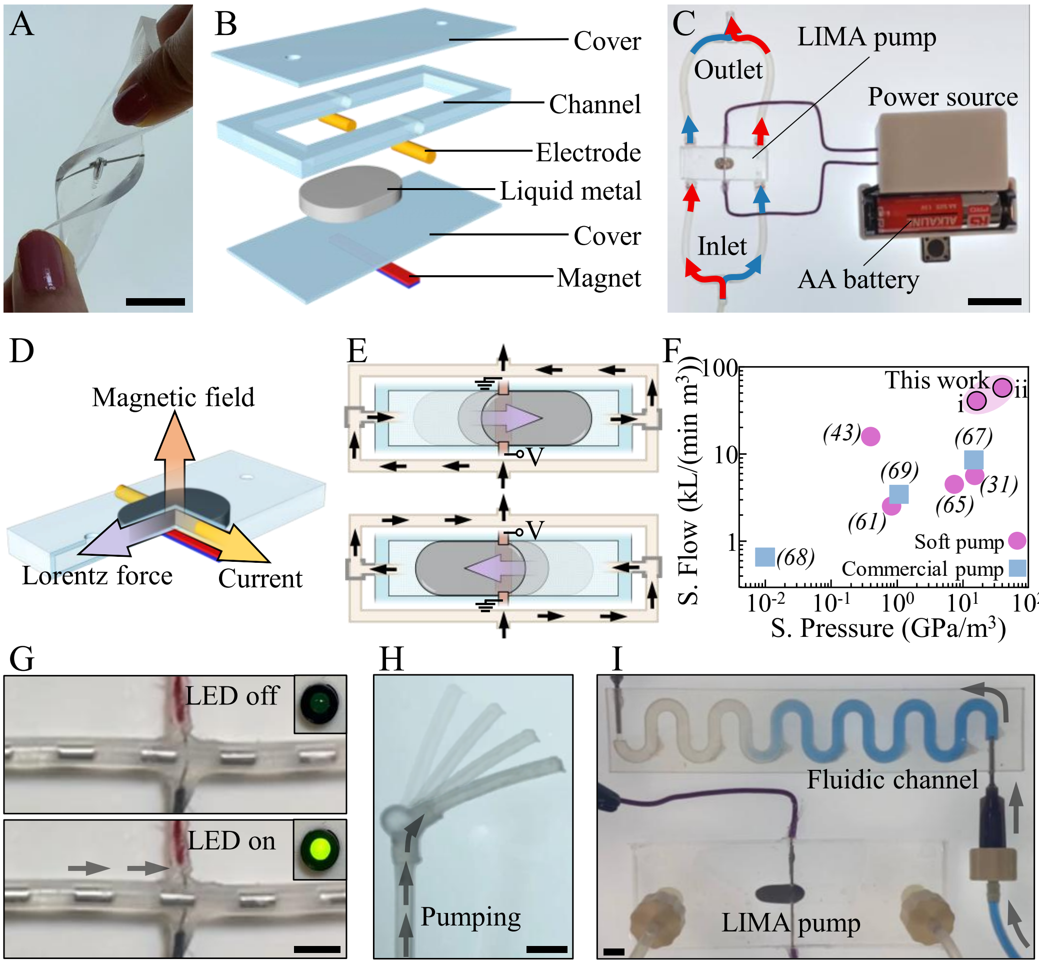

Fig.1. Principal concepts of the LIMA pump. (A) Flexibility of the LIMA pump. (see Supplementary Video S1 for full demonstration). (B) Exploded diagram of the deformable pump showing its components. (C) Demonstrating the portability and compactness of the LIMA pump. (D) The right-hand rule linking magnetic field, current density and Lorentz force acting on the liquid metal. (E) Operating principle of the pump. (F) Comparison of specific flowrate versus specific pressure of LIMA pump (solenoid (ii) and passive check valves (i)) and other soft and commercial pumps. The detailed data are presented in Table S1. (G) Control and information coding capabilities of the LIMA pump. (H) Soft actuator driven by the LIMA pump pressure. (I) Deformable LIMA pump-driven fluid flow and colour display. Scale bars are 5 mm.

Page 8: Moreover, we evaluated the system's performance by replacing the two active solenoid valves with four (non-optimal) passive check valves. Compared to the original setup, using check valves caused a small performance reduction: the maximum flow rate dropped to 3.3 ml/min and the maximum pressure to 1.6 kPa. This decrease occurred because check valves require pressure to open, as detailed in Fig. S15 and S16.

Revision to Materials and Methods:

Page 15: We also investigated the effect of using four passive check valves (AirLogic, US) on the LIMA pump's performance. The check valves were connected in pairs on both sides of the pump outlets. The maximum block pressure and flow rate of the system are demonstrated in Fig. S16. To measure cracking pressure (Fig. S15), a pressure sensor is connected between the pair of check valves on one side of the pump, with the pump outlet open.

Revision to the SI:

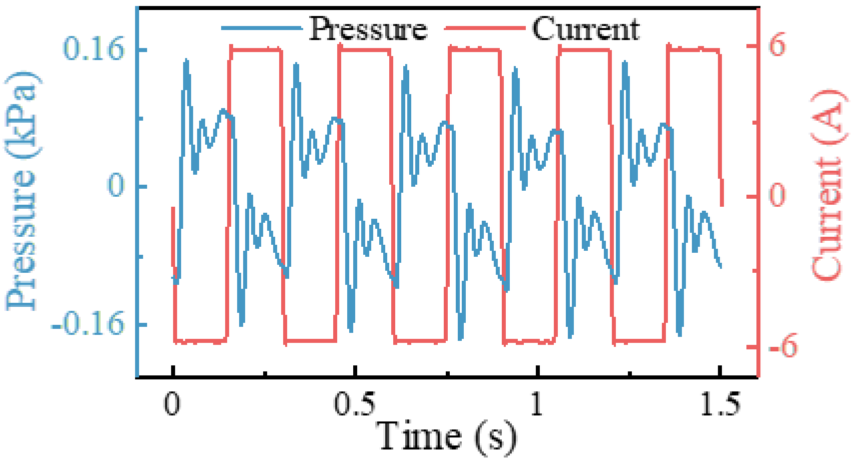

Fig S15. Measured pressure and applied current to the LIMA pump. The pressure sensor was positioned between the two check valves on one side of the pump to measure the rectified pressure. The square-wave current drives cyclic actuation of the liquid metal droplet, while the resulting periodic pressure response demonstrates unidirectional pumping enabled by the check valves, with superimposed oscillations induced by check-valve dynamics and fluid inertia.

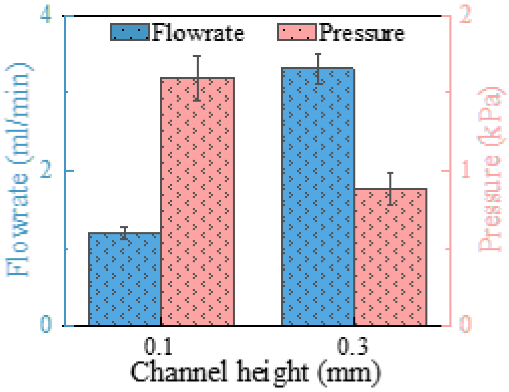

Fig S16. The maximum measured block pressure and flow rate of the LIMA pump utilising four passive check valves for the samples with heights of 0.1 mm and 0.3 mm, with an applied current of 8 Amps.

Table S1. Comparison of pump performance with leading soft and commercial pumps

Mechanism	Ref.	Mass (gr)	Vol (cm^3)	Max power consumption (W)	Max pressure (kPa)	Max flowrate (ml/min)	Pressure/ size (GPa/m^3)	Flowrate/ size (kl/min m^3)
				Soft pumps				
MHD	This work	0.2	0.086	0.8	a) 3	4.2	34.8	49.3
					b) 1.6	3.3	18.6	38.4
EHD	*(31)*	1	1.17	0.17	14	6	12	5.13
EHD	*(65)*	1.2	10.05	0.71	80	45	7.96	4.5
MHD	*(43)*	30	22.7	0.17	8	320	0.35	14
DEA	*(61)*	13.5	15	3.3	12.5	39	0.83	2.6
Magnetic	*(66)*	12.8	11.6	4	0.001	134	0.0001	11.5
				Commercial pumps				
MGD 1000S	*(67)*	142	58.6	30	800	500	13.7	8.5
McMaster STPAC	*(68)*	15422	75500	1200	1034	42500	0.01	0.56
Gotec ESX-04	*(69)*	35	24.6	5	30	83.3	1.2	3.38

Note:
a) Pumps incorporated with solenoid valves.
b) Pumps incorporated with check valves.
Acronyms: MHD, EHD, and DEA represent magnetohydrodynamics, electrohydrodynamics, and dielectric elastomer actuators, respectively.

4. *Minor issues:*

- The data in Fig. 2(H) appear discontinuous and distorted.

Response:

Thank you for the comment. We have double checked our original data in Figure 2H and believe the presented data are correct. The observed discontinuity and distortion may rise from the low quality of figure. We have improved the quality of Fig. 2 in our revision for better presentation.

Revision to the manuscript:

Revised Figure 2:

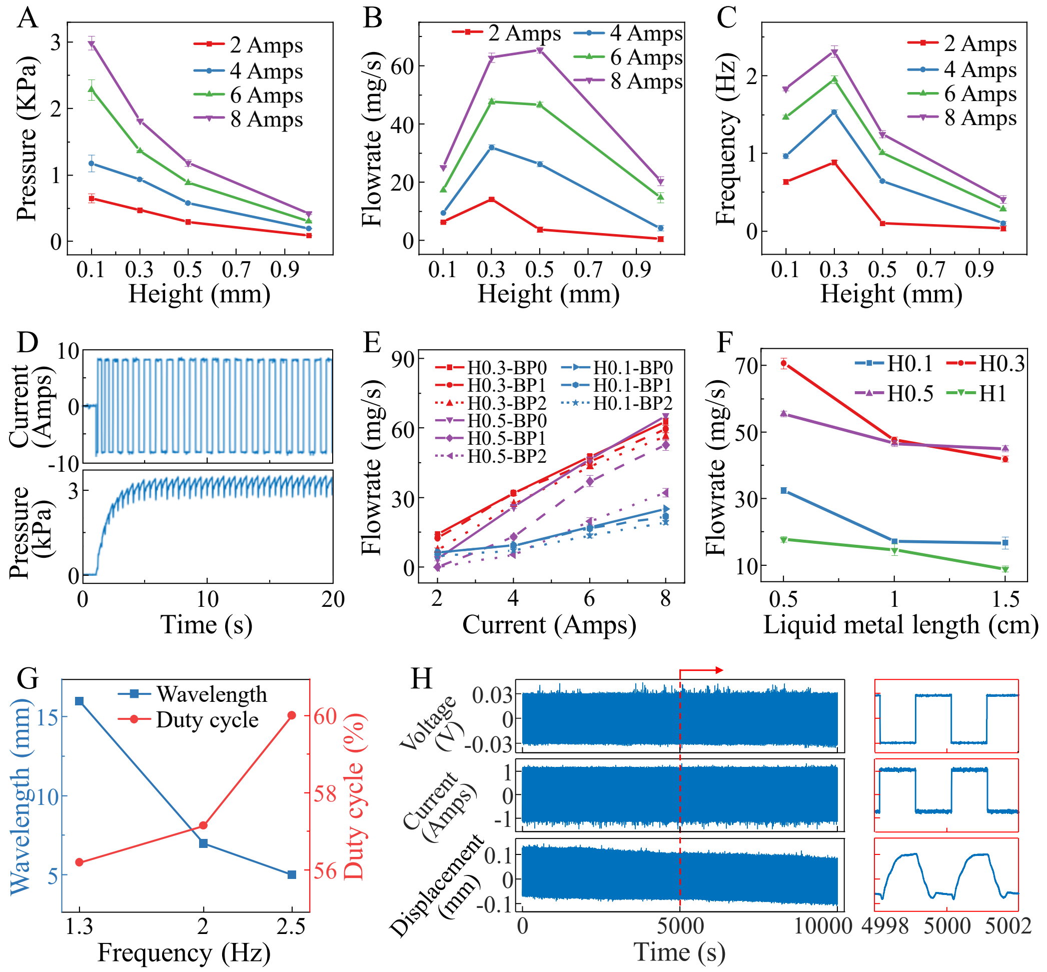

- The signal traces in Fig. 3(D) seem to be hand-drawn; actual recorded signals should be used instead.

Revision to the manuscript:

Response:

We thank the reviewer for this comment. The data shown in the original figure were smoothed, which may have given the appearance of being hand-drawn. We have now replaced the smoothed curves with the original data. The manuscript has been revised accordingly.

Revision to the manuscript:

Figure 4D:

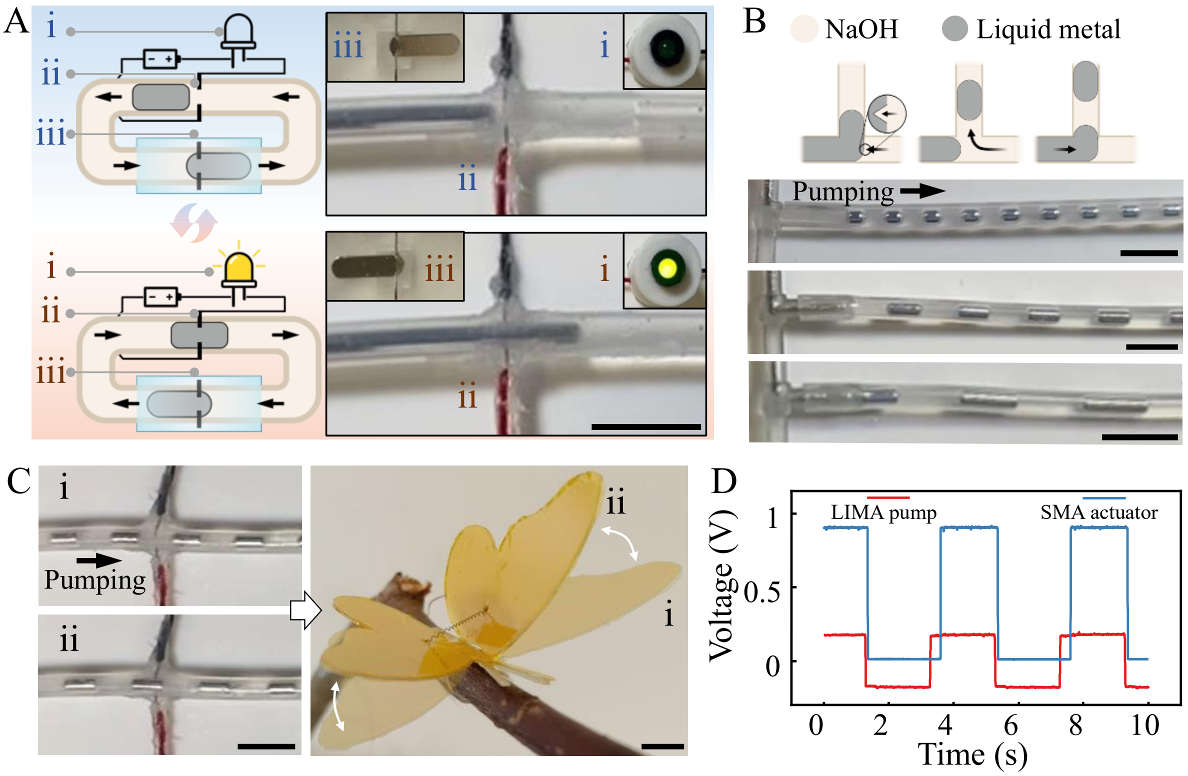

- Ref. 45 and 46 are missing.

Response:

We thank the reviewer for their comment. We have corrected the references in the manuscript.

- *The reference numbers cited in Table S1 do not correspond to the entries in the references.*

Response:

We thank the reviewer for this comment. We have corrected and updated Table S1. Please see our revised Table S1 in our previous response.

Reviewer #2 (Remarks to the Author):

This manuscript, titled "A flexible liquid metal magnetohydrodynamic pump for soft robotic systems," presents the design, characterization, and validation of a soft mechanical pump actuated by a movable liquid metal droplet. The actuation mechanism is based on Lorentz forces generated when electric current passes through the liquid metal. The pump is soft except for a small embedded magnet beneath the droplet. Three demonstrations are included to illustrate the potential applications of the pump: driving a macroscale soft actuator, converting chemical energy to mechanical energy, and generating varied code patterns for information transfer. The reviewer has the following comments and questions.

1. Claims of advantages over existing technologies are not convincingly supported.

The liquid-metal-based pump is an interesting concept. However, the demonstrations do not clearly establish the advantages or practical benefits over existing pumps.

In the Abstract, two main limitations of existing systems are described as "portability" and "system complexity." It is not evident that the proposed pump effectively addresses either:

(a) Portability:
The pump is largely soft and operates at a lower voltage, which could theoretically improve portability. However, the manuscript does not provide characterization, comparison, or evidence that demonstrates enhanced portability relative to existing devices.

(b) System complexity:
The main figures and text present the pump as a simple system. However, details in the Supplementary Information suggest otherwise. For example, the setup in Fig. S19 for the Fig. 5 experiment includes multiple valves and branches. This does not account for off-board peripherals such as the power supply or fluid sources. As presented, there is no direct evidence that the overall system is less complex than existing alternatives.

Response:

We thank the reviewer for their constructive comments. In the abstract, 'portability' and 'low complexity' refer to the simple structural components of LIMA pumps compared with their counterparts. We acknowledge that these features have not been clearly demonstrated. To bridge this gap, in our revision, we fabricated a centimetre-sized LIMA pump system. The pump is built with one-way valves to achieve directional pumping and is powered by an AA battery through a small electronic control board. The system is compact and stand-alone, showcasing the portability and low complexity of the system. In addition to the potential for further miniaturization of electronic components, the simple structure of the LIMA pump enables further miniaturization, representing a significant advantage over comparable systems. Fig.S19 shows the possibility of using a single pump to drive three different haptic cues. Here the phrase 'low complexity' is used to describe the simple structure of the pump,

instead of the overall system. The design in Fig.S19 is intended to demonstrate the pump's capabilities rather than to emphasize simplicity in system integration. When a single pump is used to perform multiple tasks, a higher level of system complexity is unavoidable. The overall system complexity can be reduced by employing multiple pumps, with each pump assigned to one task. The following edits have been applied to the manuscript to clarify these points.

Revision to the manuscript:

Page 8: As shown in Figure 3, the LIMA pump is implemented as a compact, self-contained system powered by AA batteries with integrated control electronics, enabling stand-alone and portable operation.

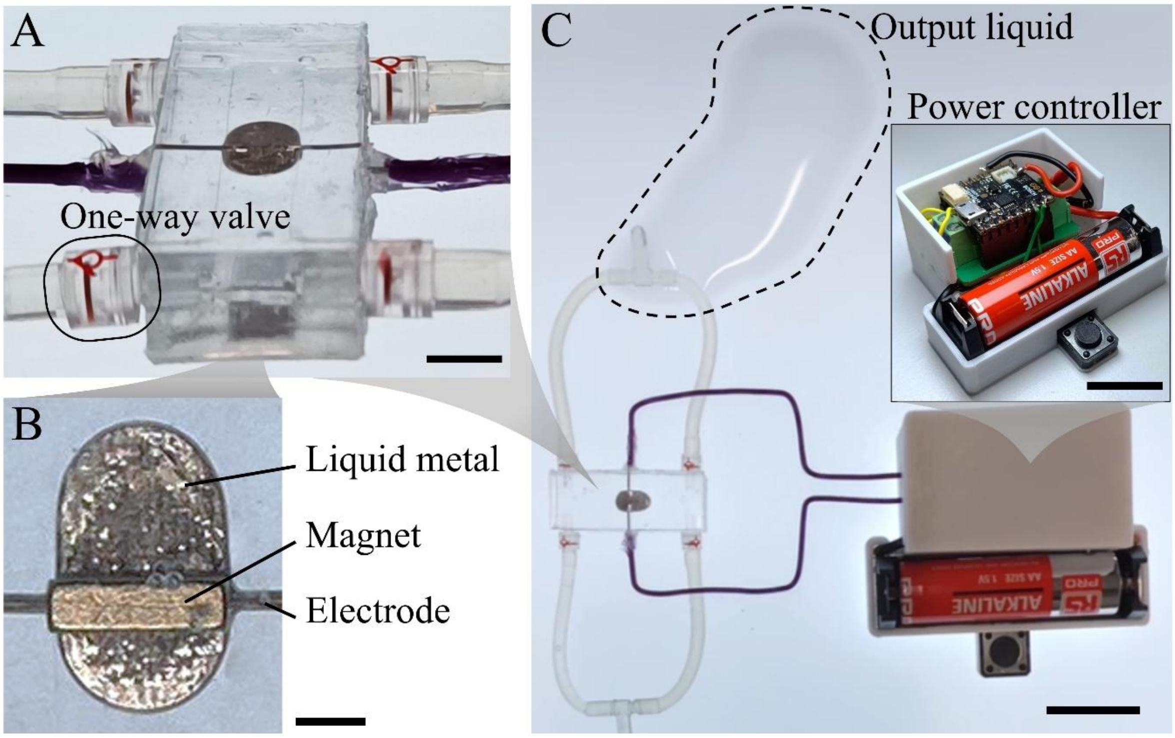

Figure 3. A portable LIMA pump system. (A) Picture of the LIMA pump integrated with four one-way valves. Scale bar is 5 mm. (B) Enlarged view of the active section of the pump. Scale bar is 2 mm. (C) The final frame of the Supplementary video 9, which shows the overview of the LIMA pump system and the displaced fluid from the output. The inserted picture shows the power controller of the pump, composed of an electronic controller, an AA battery, and a switch. Scale bar is 2 cm.

Revision to the Materials and methods:

Page 15: To reduce system complexity, four check valves (AirLogic, US) were integrated on the sides of the microchannel to enable directional pumping. The MHD actuation mechanism allows the pump to be powered by a single AA battery. The oscillation frequency of the liquid metal was controlled using a small electronic control circuit, with the potential for further miniaturization. A power switch was installed to control the on/off operation.

The electronic control circuit utilizes an Arduino Nicla Sense ME microcontroller for logic operations, powered by a single lithium coin cell battery and interfaced with a custom-built H-bridge circuit. The H-Bridge acts as the switching stage for a secondary, independent power source—a single 1.5V AA alkaline battery—which is connected directly to the pump. The Arduino directs the current from the AA battery through the pump, enabling unidirectional flow.

Supplementary information:

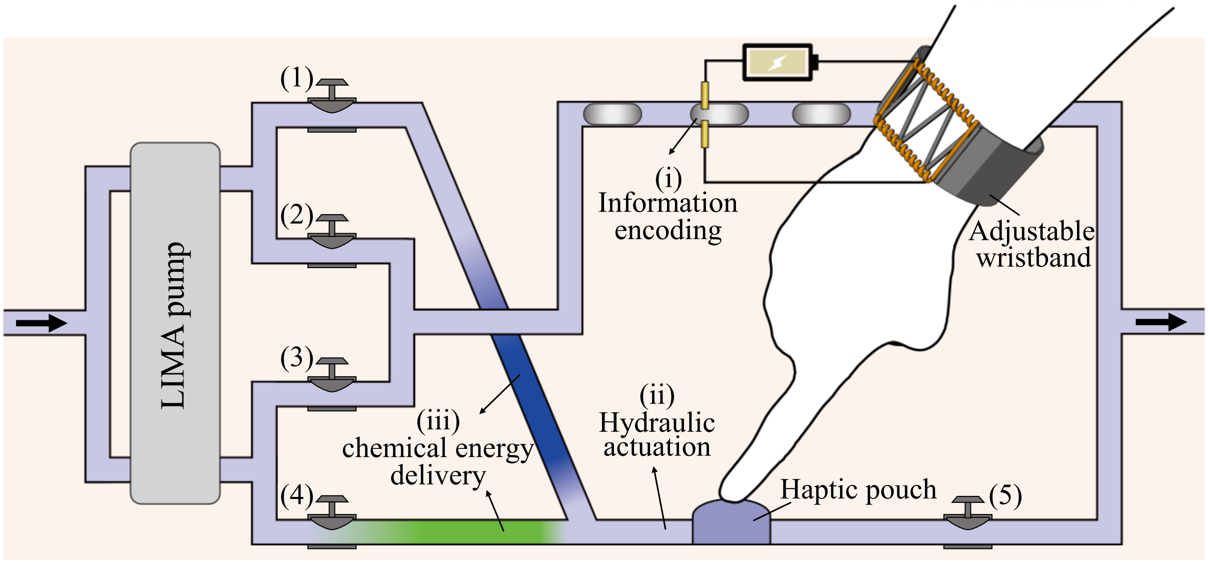

Figure S19: Schematic diagram of the integrated haptic device demonstration using a single LIMA pump to deliver three distinct functional modes sequentially: (i) information encoding, (ii) hydraulic actuation, and (iii) chemical energy delivery. A coded liquid metal signal activates the wristband to apply circumferential force in the first mode. In the second mode, the user senses a gentle pressure on their finger via hydraulic actuation. In the third mode, a higher pressure is generated by gas production from the chemical reaction. This setup demonstrates the integration of three network-like modalities within a single system. Each of these modalities can be directly driven by a separate LIMA pump to avoid system complexity. In this demonstration, valves were used to manually control the timing of each mode to avoid sensory overlap. For the first mode, valves 1 and 4 are closed, and 2 and 3 are open, to generate the code pattern and then pump the code to activate the circuit of the SMA-driven wristband. For the next mode, the 2, 3, and 5 are closed, and 1 and 4 are open to actuate and pressurise the actuator on the finger by inflating it using the hydraulic actuation. For the last mode, mode 5 is first opened for the two chemicals to reach close to the mixing point, and valve 5 is closed for the third mode chemical reaction actuation.

2.In addition, several demonstrations appear overstated relative to the preliminary nature of the results:

(a) The spider robot shown in Fig. 1A is not directly supported by the experiments in the manuscript. It is unclear how the proposed pump could feasibly enable such a system. Given the available volume in that hypothetical robot, more established rigid pumps could be used. The figure raises questions about relevance and practicality. The reference to "chromatophores" is also unclear and not integrated elsewhere in the manuscript.

Response:

Thank you for your comments. We have removed Figure 1A as it may have overstated the pump's capabilities. We also reformatted the figure for clarity and organization. Please see the revised version below.

Revision to the manuscript:

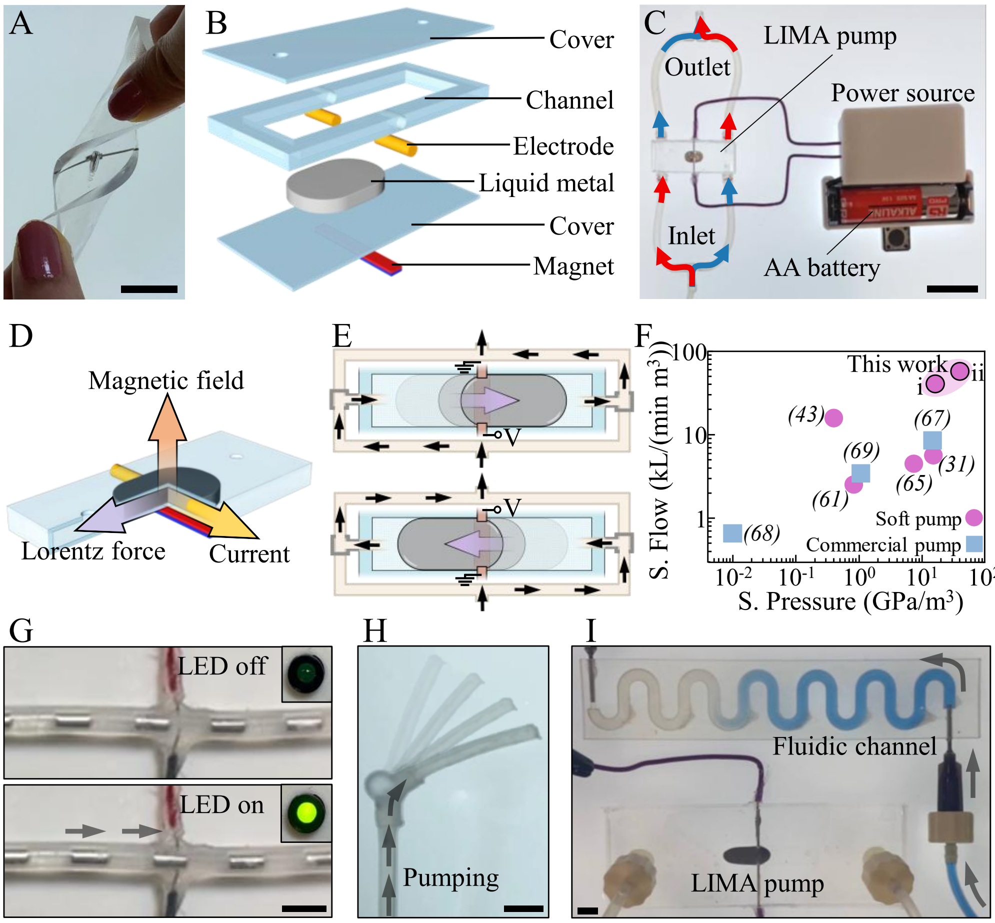

Fig.1. Principal concepts of the LIMA pump. (A) Flexibility of the LIMA pump. (see Supplementary Video S1 for full demonstration). (B) Exploded diagram of the deformable pump showing its components. (C) Demonstrating the portability and compactness of the LIMA pump. (D) The right-hand rule linking magnetic field, current density and Lorentz force acting on the liquid metal. (E) Operating principle of the pump. (F) Comparison of specific flowrate versus specific pressure of LIMA pump (solenoid (ii)

and passive check valves (i)) and other soft and commercial pumps. The detailed data are presented in Table S1. (G) Control and information coding capabilities of the LIMA pump. (H) Soft actuator driven by the LIMA pump pressure. (I) Deformable LIMA pump-driven fluid flow and colour display. Scale bars are 5 mm.

(b) Referring to the bracelet as a "display" may overstate its function.

Response:

Thank you for this helpful comment. We have revised the manuscript to use more precise wording, describing the bracelet as a soft fluidic bracelet with embedded channels used for visualisation and fluid transport. We also clarified the bracelet's colour change by demonstrating the CIE 1931 colour gamut of the three colours in an additional figure.

Revision to the manuscript:

Page 11: Fig.4. (B) A colour-changing bracelet powered by LIMA pump. i: before color change, ii: color changed in 28 seconds.

Page 12: Next, we demonstrated the capability of the LIMA pump to simultaneously pump different fluids through its differential outlet system. We fabricated a flat-helix-structured soft bracelet with two fluidic channels, each connected to an outlet of the LIMA pump via tubing. (Figure 5B). By pumping two differently colored liquids into the bracelet, the bracelet's color can be dynamically changed in 28 seconds (Supporting Information Fig. S18 and Video S6), revealing the potential for the development of customizable fashion wearables. Additionally, the dual liquid pumping system can be utilized to transfer chemical reactions. As shown in Figure 5C, a red-dyed acid (citric acid) and a blue-dyed alkali (sodium bicarbonate solution) are simultaneously injected into the helical-structured fluidic channels using a LIMA pump, and they mix at the channel outlet. The outlet is connected to a pneumatic haptic actuator. A neutralization reaction is initiated when the two liquids are mixed, producing CO2 gas that subsequently inflates the pneumatic actuator. This demonstration illustrates that the LIMA pump can serve not only as a fluid transport and color display system, but also as a novel approach for transferring chemical energy to energize remote soft actuators.

Revision to the supplementary information:

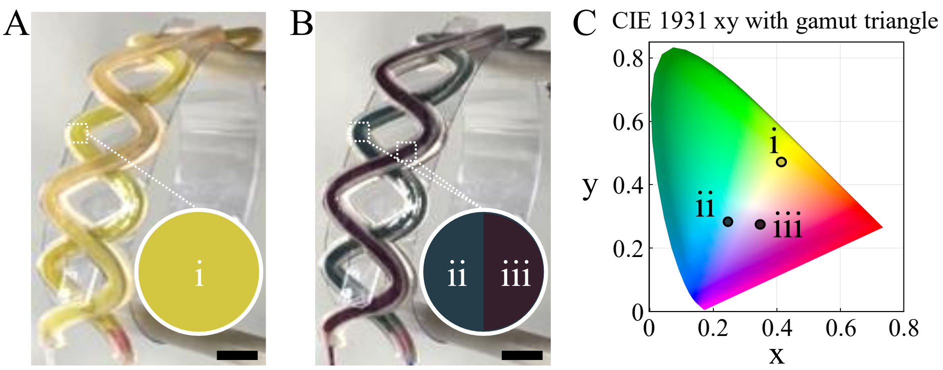

Fig S18. Color change of the bracelet. A) The color before fluid injection. B) Colors after fluid injection. C) The CIE 1931 colour gamut of the three colours of the bracelet. (i-iii) in (A-C) are matched. White balance normalisation was applied to images in (A) and (B). Scale bars are 5 mm.

(c) The term "information encoding" is used to describe control of an LED or a shape memory alloy via droplet distribution. While this may be technically defensible, it is not clear that the demonstrations meaningfully qualify as information encoding. The butterfly example in Fig. 3C does not appear to add substantive insight; its relevance to pump design or function is not explained.

Response:

Thank you for the comment. We realize the term "information encoding" may not have been sufficiently explained, and have clarified this in the revised manuscript. In this work, we have drawn inspiration from biological signalling in the body, in which chemical messengers, such as hormones, are released into the bloodstream, spread through the system, and exert an effect only when detected by the appropriate receptor, which then triggers a local response. In our case, information is carried in the spatial and temporal structure of the fluid distribution generated by the pump. As this pattern moves through the soft body, it can be detected by specific receptor elements and converted into a localized output. The resulting electrical signal can then be used to drive secondary actuators, such as LEDs or shape-memory alloys, without requiring direct fluidic actuation at the output location. The butterfly example in Fig. 3C is intended as a simple visual readout of the frequency and conductive pattern produced by the pump, rather than as a functional component of the pumping mechanism itself. The same principle is employed in the glove demonstration, in which the transmitted signal is used to generate a circular compression stimulus around the wrist. We have revised the text to clarify these points and better explain their relevance.

Revision to the manuscript:

Page 4: The growing demand and enthusiasm for soft robotics systems have motivated the development of soft computational systems to support advances in soft bodies. These systems require mechanisms for generating and transmitting control signals. Rothemund et al. (51) and Drotman et al. (52) demonstrated that periodic motion can be achieved by using a constant source of compressed fluid and the logic output from a soft bistable valve system. Garrad et al. (53) demonstrated soft matter computers to encode information and integrate computation directly into soft materials. Within this framework, our LIMA pump can perform spatiotemporal signal encoding via controlled fluid distribution, while simultaneously transferring information and energy around a soft body. We generate diverse digital code patterns by controlling the pump, with analog quantities optionally encoded in the duty cycle. Here, information is carried by the spatial and temporal structure of fluid pulses as they propagate through the body, analogous to biological signalling processes in which transported chemical messengers elicit a response only when detected by an appropriate receptor. This information is transmitted within the body of the soft robot at variable speeds to receptors, which then activate distal actuators. In the present work, these transmitted patterns are

converted into electrical signals to drive secondary elements (e.g., LEDs or shape-memory alloys), serving as readable outputs of the underlying fluidic signal without requiring direct fluidic actuation at the output site; in future implementations, similar principles could also be realized using distinguishable immiscible fluids (for example, color-coded phases) to carry information through the body. This unique combination of capabilities further distinguishes the LIMA soft pump from previous research in the field, as it couples signal generation, transmission, and fluid propulsion within a single compact unit, merging the functions of information processing (representative of neuro receptors and neurons in the brain and central nervous system) and fluid propulsion (reminiscent of the heart) within a unified, compact design. The LIMA pump is expected to enable a wide range of softer, simpler, and more intelligent soft-robotic systems (Figure 1).

Page 10: Encoded streams of liquid metal/NaOH were generated and delivered by the LIMA pump through a tube to a remote electrical switch receptor, which was connected to a shape memory alloy-actuated butterfly robot (Figure 4C). When a liquid metal droplet bridged the receptor, the butterfly's wings elevated; when a NaOH droplet passed through the receptor, the butterfly's wings relaxed. Modulating the rate and duty cycle of the generated vascular code (liquid metal/NaOH) enabled control over the wing flapping pattern (Figure 4D and Supplementary Video S3). This demonstration provides a visual readout of both the fluidic frequency and the conductive pattern produced by the pump.

Page 12: Furthermore, we created a tactile glove-like device that uses a single LIMA pump unit to deliver various tactile sensations (Figure 6C, S19). This was achieved by information transfer, hydraulic actuation, and chemical energy delivery. Specifically, the device includes a wristband that applies circumferential force to the user's wrist using integrated shape memory alloy actuators (SMAs). The code generated by the LIMA pump controls SMA actuation, demonstrating the programmable electrical switching capability of our system, as shown in Figure 6A.

3. Additional clarification is needed for certain experiments.

(a) For the experiment in Fig. 4A, the bidirectional operation of the pump is not clearly explained. How is flow direction controlled?

Response:

We thank the reviewer for their insight. To achieve bidirectional operation, we simply reverse the power supply polarity while keeping the same control system. In this way, the direction of pumping changes as the droplet's motion relative to the access to the inlet and outlet created by the valves. To clarify, we have added new text to the manuscript.

Revision to the manuscript:

Page 10: Figure 4A shows one-joint (i) and two-joint (ii) fluidic legs, inspired by the morphology of arthropod joints (*56*) and powered by the bidirectional LIMA pump achieved by switching the polarity of the power source, which generates reciprocal motion through the inflation/deflation of its soft joint(s) (see Supplementary Videos S4, S5).

Revision to the Materials and Methods:

Page 15: To achieve bidirectional pumping, we can switch the polarity of the connected power source and, by keeping the same control system for the solenoid valve, reverse the pumping direction.

(b) For the experiment in Fig. 5, the control method for the three operation modes (manual vs. automatic) should be described. The text mentions "participants," but only one individual is shown. Details such as the number of participants, experimental procedure, and number of trials per participant should be provided.

Response:

Thank your insightful comment. We have revised the manuscript to clearly describe the control method for the three operation modes shown for the tactile glove demonstration. Moreover, the tactile glove is shown as a proof-of-concept demonstration of the pump's operational modes rather than a statistically powered user study. We have edited the text to explicitly state that the experiment was conducted with a single participant, replacing the previous plural term. We acknowledge that a larger number of participants (typically on the order of 12–15, depending on effect size) would be required for statistical analysis; however, such an evaluation is beyond the scope of the present work and will be addressed in future studies.

Revision to the manuscript:

Page 12: The wearable glove-like device also includes a haptic actuator that, when worn on the finger, can deliver a gentle pressure sensation when driven by the hydraulic model of the LIMA pump and a stronger sensation when driven by the system's chemical energy delivery mode, as shown in Figure 6A. The chemical energy delivery mode in its current implementation is single-use, which could be seen as an alert sensation. The SMA and haptic displacements regarding the three sensations are shown in Figure 6B. To demonstrate the effectiveness of this system, we fitted the wearable device to the right hand of a blindfolded participant. The user was asked to press the button corresponding to each sensation they felt, based on the button's position relative to their left hand, as shown in Figure 6C. The user successfully identified the three tactile modes shown in Supplementary video S8. In this demonstration, to avoid sensory overlap, the timing of each mode was controlled manually using three-way valves (Fig. S19).

Revision to the supplementary information:

Page 19:

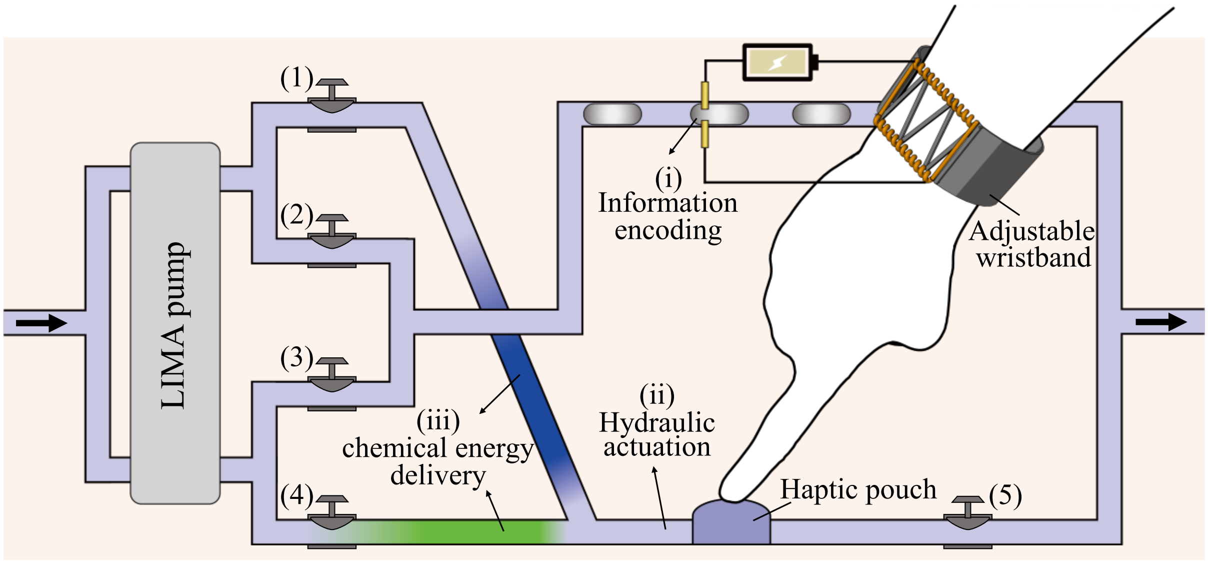

Figure S19: Schematic diagram of the integrated haptic device demonstration using a single LIMA pump to deliver three distinct functional modes sequentially: (i) information encoding, (ii) hydraulic actuation, and (iii) chemical energy delivery. A coded liquid metal signal activates the wristband to apply circumferential force in the first mode. In the second mode, the user senses a gentle pressure on their finger via hydraulic actuation. In the third mode, a higher pressure is generated by gas production from the chemical reaction. This setup demonstrates the integration of three network-like modalities within a single system. Each of these modalities can be directly driven by a separate LIMA pump to avoid system complexity. In this demonstration, valves were used to manually control the timing of each mode to avoid sensory overlap. For the first mode, valves 1 and 4 are closed, and 2 and 3 are open, to generate the code pattern and then pump the code to activate the circuit of the SMA-driven wristband. For the next mode, the 2, 3, and 5 are closed, and 1 and 4 are open to actuate and pressurise the actuator on the finger by inflating it using the hydraulic actuation. For the last mode, mode 5 is first opened for the two chemicals to reach close to the mixing point, and valve 5 is closed for the third mode chemical reaction actuation.

4. Limitations of the Fig. 5 demonstration should be acknowledged.

The third operational mode in Fig. 5 appears to be single-use only. This limitation should be clearly stated in the manuscript.

Response:

We appreciate the reviewer's observation. We have revised the manuscript to clearly state the operational characteristics of the haptic glove demonstration shown in Figure 5. More specifically, we have now noted that the third mode, associated with chemical reaction and energy transfer in its current implementation, is single-use, and in future work we will investigate alternative control strategies and coding patterns to enable repeatable operation of this mode.

Revision to the manuscript:

Page 12: The wearable glove-like device also includes a haptic actuator that, when worn on the finger, delivers a gentle pressure sensation if driven by the hydraulic model of the LIMA pump. It produces a stronger sensation when driven by the system's chemical energy-delivery mode, as shown in Figure 6A. The current implementation of chemical energy delivery is single-use and could be used as an alert signal.

Page 16: For chemical energy delivery, we used 0.9 M sodium bicarbonate (Sigma-Aldrich, UK) in water as the alkali. For the acid, we used 1.3 M citric acid (Sigma-Aldrich, UK) in water. To distinguish the solutions, we added liquid colour dyes (Honeyberry International LLP, UK). The combination of code patterning using reactive fluids for the energy transfer mode will be studied in future work.

Responses to reviewers' comments

We very much appreciate the efforts and strong positive evaluations of the editor and reviewers of our paper. We have revised the manuscript based on the constructive comments of the reviewers. The comments from referees are reprinted in *blue italic*, and our responses are marked in black. Changes made to the manuscript are marked with red.

Here, we summarize the changes we made to the manuscript in response to reviewers' comments:

1. We revised the manuscript by refining the terminology to refer specifically to the pump's mechanical simplicity and by clarifying that the electrical circuit is presented as an example implementation.
2. We revised the manuscript by reducing emphasis on signal encoding and computational functionality, clarifying their proof-of-concept nature, and refocusing on the LIMA pump as the primary contribution.
3. We revised the manuscript to clarify the preliminary nature of the haptic experiment, presenting it as a proof-of-concept demonstration rather than a validated human–robot interaction study

Reviewer #1 (Remarks to the Author):

The authors have alleviated my concerns and corrected the identified errors. The manuscript is now in a much stronger position and can be recommended for publication.

Response:

The authors thank the reviewer for their helpful comments, which have significantly improved the manuscript.

Reviewer #2 (Remarks to the Author):

The reviewer thanks the authors for making substantial efforts to address the concerns raised in the previous round of peer review. In particular, the removal of the spider robot figure and the addition of a physical, untethered prototype in the new Fig. 3 significantly strengths the manuscript. And the newly added details and explanations about the experiments are also appreciated. While now the manuscript is notably more solid, some of the underlying issues regarding the preliminary nature of the demonstrations remain. The concepts of this work is very clear, but the practical execution is still mostly rudimental and proof-of-concept.

1. Portability and complexity

The newly added standalone pump system (Fig. 3) demonstrates that the pump can operate in an untethered mode. This addition addresses the portability concern. However, the claim of "low complexity" remains. To achieve this portable operation, the system requires four one-way valves, a custom H-bridge, an Arduino Nicla Sense ME microcontroller, a lithium coin cell, and an AA battery. Even though the pump itself is structurally simple, the peripheral hardware required to make it function is not less complex than what is required for existing miniature pumps

Response:

We thank the reviewer for their comment. To clarify, our work focuses on the mechanical design of the pump, not its power supply or control electronics. By describing the system as "low complexity," we refer specifically to its mechanically simple structure: it consists of a few components, is simple to fabricate, and integrates at low cost. To avoid confusion, we have replaced the term "low complexity" with "mechanically simple" in the manuscript. Building on the mechanical simplicity, the electronic circuit in Fig. 3 is one example of standalone operation. The driving circuitry only needs a simple, low-cost oscillating signal, which can be made with a basic two-transistor circuit. The system's one-way flat valves can be easily added to the microfluidic channel design [1]. Furthermore, the fabrication cost of the actuation unit of the LIMA pump prototype is less than 50 UK pence (63 US cents), and this cost can be significantly reduced through mass production. To address the reviewer's comments, we have edited and clarified the above points through the following revisions to the manuscript.

[1] Yang, B. and Lin, Q., 2005, January. Planar Microfabricated Check Valves Utilizing Large Compliance of PDMS. In *ASME International Mechanical Engineering Congress and Exposition* (Vol. 4224, pp. 737-740).

Revision to the manuscript:

Abstract:
Fluidic soft actuators enable safe, adaptable human-machine applications, but suffer from shortcomings of portability and autonomy. Herein, we introduce a liquid metal droplet based fluidic soft pump, which is lightweight (0.2 g) and compact (0.086 cm^3), delivering specific pressure (18.6 -34.88 GPa m^{-3}) and specific flow rate (38.4 - 49.29 kl min^{-1} m^{-3}), surpassing existing soft and conventional pumps, and operating at low voltage (<1 V). Electrical energy

is converted into fluid motion through the principle of magnetohydrodynamics, which harnesses the Lorentz electro-magnetic force through the liquid metal. We present the optimization of the pump design, evaluate its fundamental characteristics, and demonstrate its integration with a range of soft robotic systems. The versatility of the pump, including powering soft actuators, transferring chemical energy, and generating diverse code patterns, illustrates the potential for the development of future advanced robotic systems.

Page 9: As shown in Figure 3, the LIMA pump is implemented as a compact, self-contained system powered by AA batteries with integrated control electronics, enabling stand-alone and portable operation. The fabrication cost of the actuation unit of the LIMA pump prototype is less than 50 UK pence (63 US cents), and this cost can be significantly reduced through mass production.

Methods:

To create a compact system, four check valves (AirLogic, US) were integrated on the sides of the microchannel to enable directional pumping. The MHD actuation mechanism allows the pump to be powered by a single AA battery. The oscillation frequency of the liquid metal was controlled using a small electronic control circuit, with the potential for further miniaturization. A power switch was installed to control the on/off operation. The electronic control circuit utilizes an Arduino Nicla Sense ME microcontroller for logic operations, powered by a single lithium coin cell battery and interfaced with an H-bridge circuit. The H-bridge serves as the switching stage for a secondary, independent power source—a single 1.5V AA alkaline battery—connected directly to the pump. The Arduino directs the current from the AA battery through the pump, enabling unidirectional flow. Future work will focus on implementing fully integrated flat valves and simplified driving electronics—for example by replacing the Arduino and H-bridge with a simple two-transistor oscillator—to further reduce system complexity and reliance on discrete components.

2. Claims about demonstrations

Defining the experiment as "spatiotemporal signal encoding" and discussing it in the context of "computational systems" and "information processing" [the newly added last paragraph of Introduction] still feel somewhat inflated considering the simple switch-closing mechanic demonstrated.

Response:

We thank the reviewer for this comment. The manuscript has been revised to clarify that these experiments are proof-of-concept demonstrations, rather than fully developed information-processing or computational systems. We have moderated the terminology accordingly and now emphasize that the primary contribution of this work is the LIMA pump itself.

Revision to the manuscript:

Page 4:

Within this system, our LIMA pump demonstrated an elementary signal encoding via controlled fluid distribution, while simultaneously transferring simple information and energy around a soft body. We generate varied digital code patterns by controlling the pump, with analog quantities optionally encoded in the duty cycle. Here, simple information is carried by the spatial and temporal structure of fluid pulses as they propagate through the body, analogous to biological signalling processes in which transported chemical messengers elicit a response only when detected by an appropriate receptor. This information is transmitted within the soft robot's body at variable speeds to receptors, which then activate the distal actuators. In the present work, these transmitted patterns are converted to electrical signals to drive secondary elements (e.g., LEDs or shape-memory alloys), serving as a proof of concept of readable outputs of the underlying fluidic signal without requiring direct fluidic actuation at the output site. In future implementations, similar principles could also be realized using distinguishable immiscible fluids (for example, color-coded phases) to carry information through the body. While these examples show the system's versatility and suggest directions for further study, the primary role of the LIMA pump is fluid propulsion, with these signal-based behaviours representing secondary capabilities enabled by its controllable flow output. The LIMA pump is expected to enable a wide range of softer, simpler, and more intelligent soft-robotic systems (Figure 1).

3. The haptic device (Fig. 6)

The reviewer thanks the authors for the clarification of the number of participants and the experiment procedure. But the newly added explanations do not change the preliminary nature of this specific demonstration. An experiment with one participant like this is a good proof-of-concept, but not a validated human-robot interaction study.

Response:

We thank the reviewer for this important clarification. We agree that an experiment involving a single participant should be considered a preliminary demonstration rather than a validated study. In response, we have revised the manuscript to more clearly reflect the scope of this work. Specifically, we avoid framing the experiment as a human–robot interaction study and instead present it as a proof-of-concept demonstration intended to illustrate the feasibility and potential of the proposed system. Our goal in this work is not to provide a comprehensive evaluation, but to demonstrate the functionality of the prototype in a controlled setting. In the revised manuscript we have emphasized the preliminary nature of the results and clarified that no generalizable conclusions are drawn from this experiment. We also highlight that a more extensive evaluation with a larger participant cohort, along with systematic optimization of the system, is planned as part of future work.

Revision to the manuscript:

Page 12: Furthermore, we created a tactile glove-like device as a proof-of-concept that uses a single LIMA pump unit to deliver various tactile sensations (Figure 6, S19). This was achieved by information transfer, hydraulic actuation, and chemical energy delivery. Specifically, the prototype includes a wristband that applies circumferential force to the user's wrist using integrated shape memory alloy actuators (SMAs). The code generated by the LIMA pump controls SMA actuation, demonstrating the simple programmable electrical switching capability of our system, as shown in Figure 6A. The preliminary wearable glove-like device also includes a haptic actuator that, when worn on the finger, can deliver a gentle pressure sensation when driven by the hydraulic mode of the LIMA pump and a stronger sensation when driven by the system's chemical energy delivery mode, as shown in Figure 6A. The chemical energy delivery mode in its current implementation is single-use, which could be seen as an alert sensation. The SMA and haptic displacements regarding the three sensations are shown in Figure 6B. To demonstrate the effectiveness of this system, we fitted the wearable prototype to the right hand of a blindfolded participant. The user was asked to press the button corresponding to each sensation they felt, based on the button's position relative to their left hand, as shown in Figure 6C. The user successfully identified the three tactile modes, as shown in Supplementary video S8. In this preliminary demonstration, to avoid sensory overlap, the timing of each mode was controlled manually using three-way valves (Fig. S19). We emphasize that this work does not constitute a validated human–robot interaction study, but rather presents an initial prototype demonstration. This kind of soft wearable haptic system can potentially provide more effective, localised, and expressive touch interactions than conventional vibrating actuators, enabling richer forms of communication, interaction, and immersion in compact wearable formats (58-60). Future work will focus on system optimization and evaluation with a larger cohort of participants.

Updated Figure 6 caption:

Figure 6: Diagram of the wearable prototype
